# A synthetic metastatic niche reveals antitumor neutrophils drive breast cancer metastatic dormancy in the lungs

**DOI:** 10.1038/s41467-023-40478-5

**Published:** 2023-08-08

**Authors:** Jing Wang, Ramon Ocadiz-Ruiz, Matthew S. Hall, Grace G. Bushnell, Sophia M. Orbach, Joseph T. Decker, Ravi M. Raghani, Yining Zhang, Aaron H. Morris, Jacqueline S. Jeruss, Lonnie D. Shea

**Affiliations:** 1 Department of Biomedical Engineering, University of Michigan, Ann Arbor, MI USA; 2 Chemical and Biological Engineering Department, Iowa State University, Ames, IA USA; 3 Department of Cariology, Restorative Sciences, and Endodontics, University of Michigan School of Dentistry, Ann Arbor, MI USA; 4 Department of Chemical Engineering, University of Michigan, Ann Arbor, MI USA; 5 Rogel Cancer Center, University of Michigan, Ann Arbor, MI USA; 6 Department of Surgery, University of Michigan, Ann Arbor, MI USA

**Keywords:** Breast cancer, Metastasis, Immunosurveillance, Cancer models, Implants

## Abstract

Biomaterial scaffolds mimicking the environment in metastatic organs can deconstruct complex signals and facilitate the study of cancer progression and metastasis. Here we report that a subcutaneous scaffold implant in mouse models of metastatic breast cancer in female mice recruits lung-tropic circulating tumor cells yet suppresses their growth through potent in situ antitumor immunity. In contrast, the lung, the endogenous metastatic organ for these models, develops lethal metastases in aggressive breast cancer, with less aggressive tumor models developing dormant lungs suppressing tumor growth. Our study reveals multifaceted roles of neutrophils in regulating metastasis. Breast cancer-educated neutrophils infiltrate the scaffold implants and lungs, secreting the same signal to attract lung-tropic circulating tumor cells. Second, antitumor and pro-tumor neutrophils are selectively recruited to the dormant scaffolds and lungs, respectively, responding to distinct groups of chemoattractants to establish activated or suppressive immune environments that direct different fates of cancer cells.

## Abstract (web summary)

3D scaffolds can be used to recapitulate key aspects of the microenvironment of primary tumors and metastatic organs. Here the authors use subcutaneous porous 3D scaffold implants as a tool to study the immune signals in the lungs of metastatic breast cancer, revealing multifaceted roles of neutrophils in regulating lung metastasis.

## Introduction

Distant metastases dramatically decrease the survival rate of breast cancer patients^1^. The formation of secondary tumors begins with early organ-tropic dissemination and colonization of tumor cells (TCs) at a metastatic niche (MN) within a distant organ^2,3^. Afterward, TCs may proliferate in an immunosuppressive environment or enter cancer dormancy in an MN with potent antitumor immunity^4^. Cancer dormancy includes the small non-expanding population of TCs resulting from the equilibrium between TC proliferation and death (tumor mass dormancy) and the non-proliferating status of TCs entering growth arrest yet remaining viable (cancer cell dormancy)^4,5^. Dormant TCs can be awakened and start growing as a decrease of antitumor immunity in the MNs years after surgically removing the primary tumor^4,5^. These metastatic events are regulated by the immune signals in the MNs^6^. Identifying the master regulators driving or inhibiting each step of the metastatic cascade is challenging yet crucial to developing the immunotherapeutic targeting the environmental regulation of metastasis to drive metastatic dormancy in major organs.

Complex signals in the MNs can be deconstructed by three-dimensional (3D) scaffolds that provide a defined site to recapitulate the key aspects of the microenvironments in the primary tumor or metastatic organs. The widely used models are in vitro 3D scaffolds seeded with tumor cell lines and stromal cells^7,8^. These scaffold models have facilitated studying how environmental factors (e.g., cytokines, extracellular matrix proteins, and environmental stiffness) influence cancer cell dormancy^7^. However, they have limitations in deciphering the mechanisms underlying immune regulation of metastasis. First, these in vitro models focus on analyzing incorporated factors while missing intrinsic signals derived from the primary tumor and tumor-educated immune system in vivo^9^, including master signals regulating different metastatic events. Second, tumor cell lines added to the scaffolds differ from TCs migrating from the primary tumor to MNs in molecular phenotypes and responses to niche signals^10^. Such a defect may limit our understanding of metastasis.

To overcome this limitation, we recently developed in vivo synthetic MN models, which were immune niches in porous 3D scaffolds subcutaneously implanted in animal models of cancer disease^11–17^. Porous 3D scaffolds were made of synthetic biomaterials that were biocompatible and degradable, such as polycaprolactone (PCL) and poly(lactide-co-glycolide) (PLG)^12,13^. The porosity of these scaffolds allowed vascular in-growth after they were implanted in animal models^18^. Multiple types of immune cells can infiltrate the scaffolds through newly developed blood vessels, establishing an immune niche in the scaffolds. The immune cell composition in the scaffolds changed significantly in the presence of the primary tumor. In mice with metastatic breast cancer, Gr1^+^CD11b^+^Ly6G^+^ neutrophils increased in scaffold implants and the major organs (e.g., lungs and spleen) as cancer progressed^12,13^. More interestingly, these cancer-induced immune alterations in the scaffold implants created synthetic MNs favorable for attracting circulating tumor cells. Accumulation of circulating tumor cells in the subcutaneous scaffold implants has been identified in multiple breast cancer models^12,13^.

In this work, we isolate breast cancer TCs seeding the scaffolds and expand them to a new cell line in vitro^14^. The characterization of this new cell line reveals its higher aggressiveness than its parental TCs in vitro and in vivo, and high similarity to lung-tropic breast cancer cells in terms of gene expression pattern^14^. These results imply that the diseased scaffolds and lungs employ a similar group of immune signals (e.g., cancer cell chemoattractants) to attract lung-tropic circulating tumor cells in animal models with metastatic breast cancer. In the first part of our study, we comprehensively investigate the immune environments in scaffolds and lungs in the absence and presence of primary tumors to identify signals conducting the organ-tropic metastatic seeding of circulating TCs in the lungs.

Although TCs isolated from diseased scaffolds and lungs share similarities and are more aggressive than their parental cells^14^, these TCs have disparate fates after they seed these two niches in vivo. We find that in metastatic breast cancer, TCs are gradually eradicated in diseased scaffolds and reach metastatic dormancy with no increase in the number of TCs in the scaffolds as cancer progresses. In contrast, TCs seeding the lungs eventually expand to lethal metastatic tumors. This finding motivates the use of the “dormant” synthetic MNs to identify immune signals regulating metastatic dormancy in breast cancer. In the second part of our study, we compare the signals in the synthetic MNs with that in the lungs derived from metastatic breast cancer models that can develop lethal secondary tumors and the lungs derived from non-metastatic breast cancer models that can recruit TCs but inhibit their outgrowth to secondary tumors.

The two parts of our study reveal that Gr1^+^CD11b^+^ Ly6G^+^ neutrophils play a key role in all three important events in breast cancer metastasis: metastatic seeding, metastatic outgrowth, and metastatic dormancy. Gr1^+^CD11b^+^Ly6G^+^ neutrophils have been recognized as an important immune regulator of cancer metastasis. However, controversy has been surrounding the pro- and antitumor roles of neutrophils^19–21^. Most previous studies focused on how neutrophils promoted tumor growth and metastasis, while only a few addressed their antitumor mechanisms at the metastatic sites, probably due to limitations in previous research methods. Here, our study of the in vivo synthetic MN model addresses the controversy about Gr1^+^CD11b^+^Ly6G^+^ neutrophils in breast cancer by revealing the pro-tumor roles of neutrophils in regulating lung-tropic metastatic dissemination of breast cancer cells and supporting metastatic outgrowth as well as their antitumor roles in boosting antitumor immunity and driving metastatic dormancy in some conditions. The multifaceted roles of Gr1^+^CD11b^+^Ly6G^+^ neutrophils, a group of heterogeneous cell populations in the cancer setting, are associated with multiple phenotypes and functions of their subgroups. Our study of Gr1^+^CD11b^+^Ly6G^+^ neutrophils reveals their multifaceted mechanisms contributing to breast cancer metastasis.

## Results

### Synthetic MNs and lungs recruit lung-tropic metastatic breast cancer cells

**Fig. 1:**
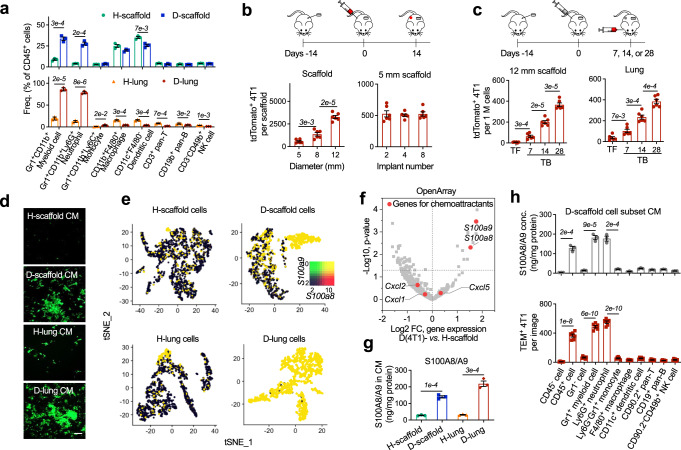
Subcutaneous scaffold implants and lungs in BALB/c mice with 4T1 metastatic breast cancer are infiltrated with Gr1^+^CD11b^+^Ly6G^+^ neutrophils that overproduce S100A8/A9 cytokines. **a** The frequencies of immune cell subsets in scaffolds and lungs derived from tumor-free BALB/c mice and mice bearing 4T1 tumor. H healthy, D diseased. **b** Tumor cells spontaneously migrate from the primary tumor to scaffolds of mice bearing 4T1-Luc2-tdTomato tumor. Scaffold implants were analyzed in the middle stage of cancer. **c** In vivo extravasation of intracardially injected 4T1-Luc2-tdTomato cells to scaffolds and lungs of tumor-free mice and mice bearing non-fluorescent 4T1 tumor at different cancer stages. The humane endpoint of BALB/c mice bearing 4T1 tumor without treatment was ~28 days after orthotopic tumor inoculation due to lethal lung metastases. TF tumor-free, TB tumor-bearing. **d** In vitro extravasation of 4T1 cells to conditioned media prepared by cells derived from tumor-free mice or mice bearing 4T1 tumor. Experiments were performed in the Boyden chamber. 4T1 cells were stained with a green dye and then placed in the top chamber while the conditioned media was placed in the lower chamber. Bar = 20 µm. CM conditioned media, TEM trans-endothelial migration. **e**, **f** *S100a8* and *S100a9* genes are overexpressed in diseased tissues relative to healthy tissues. Gene expression in these tissues were analyzed by single-cell RNA sequencing (**e**) and OpenArray (**f**). FC fold change. **g** The abundance of S100A8/A9 cytokines in media conditioned by healthy or diseased tissues. **h** The abundance of S100A8/A9 cytokines in media conditioned by cell subsets derived from diseased scaffolds (top panel) and in vitro extravasation of 4T1 cells to these conditioned media (lower panel). Data are shown as Mean ± SEM and *p* values are from student’s two-tailed *t* test. *n* = 3 for (**a**), (**g**), (**h**, top panel), and *n* = 6 for (**b**), (**c**), (**h**, lower panel). Source data are provided as a Source Data file.

To create the in vivo synthetic MNs, we subcutaneously implanted cargo-free porous PCL scaffolds in immune-competent BALB/c mice before orthotopic inoculation of 4T1 murine metastatic breast cancer cells in the fat pad^12–14^. PCL is a synthetic and biodegradable polyester polymer used in many FDA-approved implants^22^. We implanted the scaffolds before tumor inoculation to minimize the influences of scaffold implantation on the development of metastatic breast cancer models. In the middle stage of 4T1 cancer, we observed significant concurrent expansion of Gr1^+^CD11b^+^ myeloid cells in diseased scaffolds (D-scaffold or D-scaf) and lungs (D-lung) (Fig. 1a). These cells were predominantly granulocytic (Ly6G^+^), identical to neutrophils in the cancer setting^19^. Gr1^+^CD11b^+^ myeloid cells are a group of heterogeneous cell populations expanding in multiple types of cancers, which were also called myeloid-derived suppressor cells (MDSCs)^23^. Accumulating evidence has demonstrated the suppressive activity of Gr1^+^CD11b^+^ myeloid cells and their Ly6G^+^ subpopulation on tumor-killing T or NK cells, and for this reason, they were regarded as an important player in promoting cancer progression and metastasis^20,23^. In our study, we found that Gr1^+^ cells (predominantly neutrophils) isolated from diseased scaffolds and lungs at the late stage of cancer suppressed T cell proliferation in vitro while those isolated before the advanced cancer stage showed no suppression activity (Fig. S1), implying the dynamic change of Gr1^+^CD11b^+^Ly6G^+^ neutrophils with cancer progression.

Following immune alterations, we observed that D-scaffold recruited TCs from circulation, a key feature of pre-metastatic niches^24^. In BALB/c mice orthotopically inoculated with 4T1-Luc2-tdTomato cells (spontaneous metastasis model), fluorescent TCs migrated to scaffolds and lungs from the primary tumor. Their abundance per scaffold correlated with the size of the scaffolds yet was unaffected by the implant number (Fig. 1b, S2). Consistently, in the experimental metastasis model with 4T1-Luc2-tdTomato cells delivered to mice with or without non-fluorescent 4T1 primary tumors through intracardiac injection, substantially more tdTomato^+^ TCs accumulated in scaffolds and lungs of tumor-bearing mice relative to tumor-free mice. And both diseased tissues recruited more TCs as the disease progressed (Fig. 1c, S3). The contribution of the primary tumor to the formation of pre-metastatic niches was also verified in an in vitro extravasation model (Boyden chamber). Media conditioned by cells derived from diseased tissues rather than healthy ones activated trans-endothelial migration (TEM) of 4T1 cells in vitro (Fig. 1d), suggesting tissues from tumor-bearing mice produced chemoattractants for TCs, which were lacking in tumor-free animals.

**Fig. 2:**
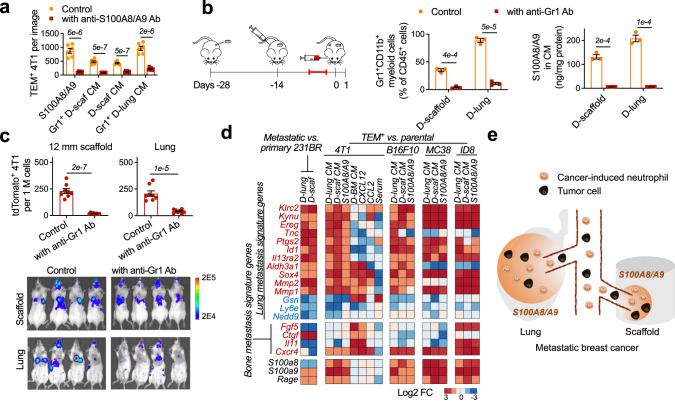
Subcutaneous scaffold implants and lungs in BALB/c mice with 4T1 metastatic breast cancer employ S100A8/A9 cytokines to recruit tumor cells with lung metastasis gene signature from the circulation. **a** Neutralizing S100A8/A9 in the conditioned media with antibodies decreased in vitro extravasation of 4T1 tumor cells to the conditioned media. Ab antibody, D-sca diseased scaffold. **b**, **c** Administration of anti-Gr1 antibodies to BALB/c mice bearing 4T1 tumor depleted the Gr1^+^CD11b^+^ myeloid cells (**b**), decreased the abundance of S100A8/A9 cytokines (**b**), and decreased the in vivo extravasation of intracardially injected 4T1-Luc2-tdTomato cells to diseased scaffolds and lungs (**c**). Antibodies were administrated at the intermediate cancer stage (marked red in the timeline). The abundance of 4T1-Luc2-tdTomato tumor cells in the tissues was measured by flow cytometry and IVIS the second day after fluorescent 4T1 tumor cells were administrated to BALB/c mice bearing non-fluorescent 4T1 tumor through intracardiac injection. **d** Expression of signature genes mediating breast cancer metastasis to lungs^28^ or bones^30^ in tumor cells transmigrating towards different cancer cell chemoattractants and conditioned media in vitro, and those colonizing diseased scaffolds and lungs in vivo. Gene signature names are to the left. Red text is overexpressed in the signature while blue text is under-expressed in the signature. 231BR MDA-MB-231BR, BM bone marrow. **e** Schematic illustration of how scaffolds and lungs recruit the same subpopulation of tumor cells from the circulation in mouse models with 4T1 metastatic breast cancer. Data are shown as Mean ± SEM and *p* values are from student’s two-tailed *t* test. *n* = 3 for (**a**), (**b**), and *n* = 6 for (**c**). Source data are provided as a Source Data file.

The S100A8/A9 protein complex^25^ was subsequently identified as the most significantly increased chemoattractant for TCs in D-scaffold and D-lung relative to healthy tissues (Fig. 1e–g). We isolated different cell populations from the diseased scaffolds (Fig. 1h-top panel) and lungs (Fig. S4) with magnetic-activated cell sorting (MACS) and analyzed their production of S100A8/A9 proteins by ELISA or qRT-PCR. And we verified that S100A8/A9 originated from granulocytic Gr1^+^CD11b^+^Ly6G^+^ neutrophils. Consistently, conditioned media (CM) prepared by scaffold-derived or lung-derived cell populations that included Gr1^+^CD11b^+^Ly6G^+^ neutrophils (CD45^+^ leukocytes, Gr1^+^ myeloid cells, or Ly6G^+^ neutrophils) all included a high amount of S100A8/A9 and activated trans-endothelial migration of 4T1 cells in vitro (Fig. 1h-lower panel). Extravasation of 4T1 cells to D-scaffold and D-lung was suppressed when S100A8/A9 in CM was neutralized *via* antibody-binding in vitro (Fig. 2a, S5) or when the abundance of S100A8/A9 in MNs decreased following the systemic depletion of Gr1^+^CD11b^+^ myeloid cells with anti-Gr1 antibodies in vivo (Fig. 2b, c)^26^. Anti-Gr1 antibodies, which reduced both Gr1^+^CD11b^+^Ly6G^+^Ly6C^−^ granulocytic myeloid cells (neutrophils) and Gr1^+^CD11b^+^Ly6G^−^Ly6C^+^ monocytic myeloid cells, were administered to mice bearing non-fluorescent 4T1 tumor three times through intraperitoneal injection. Anti-Gr1 antibodies depleted >90% of neutrophils that are the major type of myeloid cells (Fig. 1a) and 50-60% of monocytic myeloid cells that did not produce S100A8/A9 (Fig. 1h, S6). Due to the dramatic decrease in the frequencies of neutrophils in diseased lungs and scaffolds, the concentration of S100A8/A9 proteins in these niches was reduced by >95% (Fig. 2b). Consequently, diseased scaffolds and lungs in BALB/c mice bearing non-fluorescent 4T1 tumor failed to recruit 4T1-Luc2-tdTomato cells administered through intracardiac injection (Fig. 2c). These results, together with our in vitro studies, suggested that scaffolds and lungs recruited tumor cells from the circulation through S100A8/A9 secreted by neutrophils in 4T1 metastatic breast cancer.

S100A8/A9 predetermines lung metastasis of many types of cancer^27^. We found that S100A8/A9 specifically recruited TCs with a lung metastasis gene signature to the synthetic MNs and lungs. We isolated 4T1 TCs and other murine TCs (B16F10 melanoma, MC38 colon carcinoma, ID8 ovarian cancer, Fig. S7) transmigrating towards S100A8/A9-included CM within the Boyden chamber in vitro. We also isolated MDA-MB-231BR (231BR) human breast cancer cells metastasizing to S100A8/A9-abundant MNs (D-scaffold and D-lung) in vivo and then expanded them to cell lines in vitro^14^. These TC subpopulations overexpressed the genes mediating breast cancer metastasis to the lungs relative to the corresponding parental cells (Fig. 2d)^28^. In contrast, CXCL12, a chemokine promoting bone metastasis^29^, and media conditioned by diseased bone marrow (D-BM) attracted 4T1 cells overexpressing signature genes regulating bone metastasis^30^. CCL2 and serum that are non-organ-specific TC chemoattractants attracted 4T1 cells with neither signature (Fig. 2d). These results indicated that metastatic organotropism partly resulted from the selective recruitment of TCs with a similar gene expression pattern in response to the organ-specific TC chemoattractants in MNs. Diseased scaffolds and lungs in metastatic breast cancer models were abundant with S100A8/A9, and therefore both MNs recruited TCs with lung metastasis gene signature from the circulation (Fig. 2d). This finding supplemented Paget’s “seed and soil” theory of metastasis^3^ by suggesting that similar “soils” (MNs) favor the metastatic seeding of the same group of “seeds” (TCs).

In lung metastasis, the autocrine loop through S100A8/A9 and their receptor RAGE for TC recruitment were also common to the recruitment of Gr1^+^CD11b^+^ myeloid cells^25^, suggesting TCs employ the same trafficking pathway for cancer-induced neutrophils in their homing to the synthetic MNs and lungs in vivo^20^.

Overall, in the first part, we compared the immune environments in the scaffolds and lungs derived from tumor-free and tumor-bearing mice, and we also compared the gene signature of TCs attracted to diseased scaffolds and lungs. Our results indicated that, in metastatic breast cancer, Gr1^+^CD11b^+^ neutrophils expanded and infiltrated lungs and subcutaneous scaffold implants in mouse models with metastatic breast cancer. These neutrophils secreted S100A8/A9 cytokines to recruit circulating breast cancer TCs overexpressing the receptor of S100A8/A9 (Fig. 2e). The common use of S100A8/A9 to recruit TCs resulted in that TCs accumulating at the scaffolds shared similar gene signature to TCs that accumulated at the lungs^14^.

### Synthetic MNs and lungs induce metastatic dormancy and outgrowth, respectively, in metastatic breast cancer

**Fig. 3:**
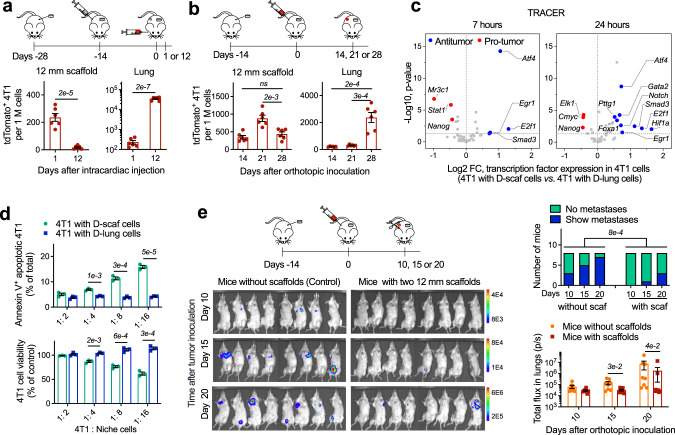
The synthetic MNs (scaffold implants) eradicate tumor cells and drive metastatic dormancy, while the endogenous lungs promote metastatic outgrowth in BALB/c mice with 4T1 metastatic breast cancer. **a**, **b** Different fates of 4T1-Luc2-tdTomato cells recruited to diseased scaffolds and lungs of BALB/c mice bearing 4T1 tumor in experimental (**a**) and spontaneous (**b**) metastasis models. In the experimental metastasis model, 4T1-Luc2-tdTomato cells were transfused to BALB/c mice bearing non-fluorescent 4T1 tumor through intracardiac injection, and then tdTomato^+^ cells in MNs were measured by flow cytometry at day 1 and day 12 after injection. In the spontaneous metastasis model, 4T1-Luc2-tdTomato cells were orthotopically inoculated to BALB/c mice, and then tdTomato^+^ cells in the MNs were quantified at days 14, 21, and 28 after tumor inoculation. **c** Antitumor and pro-tumor transcription factors are differentially overexpressed in 4T1 cells co-cultured with diseased scaffold and lung cells. Activities of transcription factors were measured by TRACER (TRanscriptional Activity CEll aRrays). **d** Apoptosis and in vitro proliferation of 4T1-Luc2-tdTomato cells co-cultured with cells derived from diseased scaffolds or lungs. **e** Lung metastases of BALB/c mice without or with scaffold implants at days 10, 15, and 20 after orthotopic inoculation of 4T1-Luc2-tdTomato, representing early, intermediate, and late stages of cancer, respectively. Mice were immediately imaged after surgically resecting the primary tumor at the desired time. Mice bearing scaffold implants were less likely to have detectable metastases than mice without implants (top panel, *p* value is from Fisher’s exact test). Mice bearing scaffolds had reduced luminescence localized to the lung in the day 15 and day 20 groups (lower panel, *p* values are from non-parametric Mann–Whitney test). Data are shown as Mean ± SEM. *p* values are from student’s two-tailed *t* test when not otherwise specified. *n* = 6 for (**a**), (**b**), *n* = 3 for (**d**, top and lower panels), and *n* = 8 for (**e**, lower panel). Source data are provided as a Source Data file.

TCs recruited to scaffolds and lungs exhibited distinct fates, despite being similar in lung metastasis gene signatures (Fig. 2d), high aggressiveness^14^, and overexpression of markers for cell stemness (Fig. S8). We administrated 4T1-Luc2-tdTomato cells to mice bearing non-fluorescent 4T1 tumors through intracardiac injection and observed their growth. On the following day after injection, a large amount of tdTomato^+^ TCs was attracted to scaffolds and lungs (~200 TCs per 1 M total cells in both scaffolds and lungs) (Fig. 3a). However, on day 12 after injection, 4T1-Luc2-tdTomato cells recruited to D-scaffold were gradually eradicated, and its number in scaffolds decreased to ~10 TCs per 1 M total cells. In contrast, those seeding D-lung expanded to ~40,000 TCs per 1 M total cells (Fig. 3a). In mice with orthotopically inoculated 4T1-Luc2-tdTomato tumor, the balance between continuous TC recruitment and death resulted in a steady number of TCs in the synthetic MNs, a status called tumor mass dormancy (Fig. 3b, days 14 *vs*. 28)^4,5^. In contrast, TCs in D-lung aggressively proliferated to lethal metastases at the late stage of cancer (Fig. 3b, days 14 and 21 *vs*. 28). In vitro real-time analysis of transcription factor (TF) activity in TCs through a technology called TRanscriptional Activity CEll aRrays (TRACER)^31^ showed that TFs regulating endoplasmic reticulum (ER) stress and apoptosis (e.g., *E2f1*) were activated in 4T1 cells co-culturing with D-scaffold cells, while TFs promoting cell growth (e.g., *Nanog*) were overexpressed in 4T1 cells co-cultured with D-lung cells (Fig. 3c). Consistently, D-scaffold cells induced 4T1 cell apoptosis and inhibited their proliferation in vitro, while D-lung cells promoted their growth (Fig. 3d), consistent with the in vivo fates of 4T1 cells.

Because the synthetic MNs recruited and eradicated a portion of TCs that would otherwise have colonized the lungs, we observed that mice bearing two scaffold implants developed lung metastases later than mice receiving a mock scaffold implantation surgery after the 4T1 primary tumors were surgically resecting (Fig. 3e). Therefore, mice with scaffolds achieved a modest survival advantage compared to mice without scaffolds (*p* < 0.05, from Mantel-Cox test, Fig. S9).

### The inflammatory responses to biomaterials developed potent antitumor immunity in the synthetic MNs

**Fig. 4:**
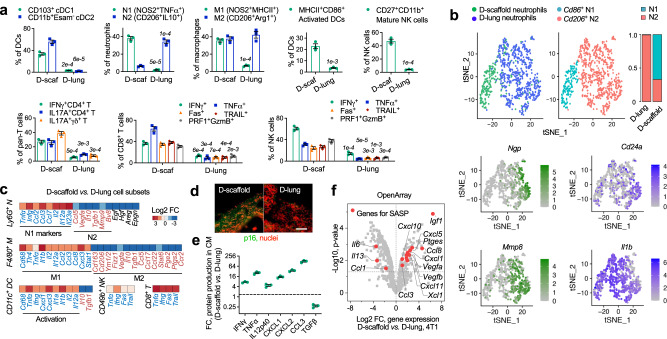
The synthetic MNs (scaffold implants) develop potent in situ antitumor immunity through biomaterial-induced inflammation in 4T1 metastatic breast cancer. **a**–**c** Phenotypic characterization of immune cells in diseased scaffolds and lungs of BALB/c mice bearing 4T1 tumor on activation and polarization markers by flow cytometric (**a**), single-cell RNA sequencing (**b**), and qRT-PCR analyses (**c**). In single-cell RNA sequencing analysis, *Cd86* (cyan) and *Cd206* (red) are the markers for N1 and N2, respectively. *Ngp* and *Mmp8* (dark green) are makers for immature neutrophils, while *Cd24a* and *Il1b* (blue) are makers for mature neutrophils^37,38^. N neutrophil, M macrophage, PRF1 perforin, GzmB granzyme B. **d**–**f** Expression of the cellular senescence marker p16 proteins (**d**, Bar = 200 µm), production of cytokines and chemokines (**e**), and expression of genes associated with the senescence-associated secretory phenotype (SASP) (**f**) in diseased scaffolds and lungs of BALB/c mice bearing 4T1 tumor. Data are shown as Mean ± SEM and *p* values are from student’s two-tailed *t* test. *n* = 3 for (**a**), (**e**). Source data are provided as a Source Data file.

Subsequently, we found that the TCs were efficiently eradicated in the synthetic MNs because scaffold implants developed potent antitumor immunity, resulting from the locally elicited immunity in response to the PCL synthetic biomaterials, termed foreign body responses (FBR)^32^. PCL-driven local pro-inflammatory responses in BALB/c mice bearing 4T1 tumor exhibited the following characteristics. First, PCL scaffolds were infiltrated with activated dendritic cells (DCs), predominantly CD103^+^ cDC1 capable of actively presenting antigens and CD11b^+^Esam^-^ cDC2 capable of efficiently priming T cells (Fig. 4a)^33,34^. Second, Th1 and Th17 responses were activated at the FBR site, reflected by high frequencies of IFNγ^+^ and IL-17A^+^CD4^+^ T cells and IL-17A^+^γδ^+^ T cells, mass production of pro-inflammatory cytokines, and polarization of immune cells towards inflammatory phenotypes (e.g., N1, M1, activated DCs, Fig. 4a–c, flow cytometric gating strategy is shown in Fig. S10)^34–36^. Note that neutrophils in the scaffolds were predominantly N1-polarized antitumor neutrophils that overexpressed markers such as *Cd86, Nos2*, and *Fas*, and most of these cells were also mature neutrophils that overexpressed maturation markers such as *Cd24a* and *Il1b*^37,38^. Third, Th17 induced senescence in cells surrounding PCL scaffolds, featured by overexpression of p16^INK4a^ proteins, a senescence biomarker, and overexpression of genes associated with the senescence-associated secretory phenotype (SASP) (Fig. 4d–f)^39^. These characteristics of FBR were the same as those observed in PCL scaffolds implanted in tumor-free BALB/c mice^40^.

These cell phenotypes or signals that were associated with foreign body responses also contributed to developing a pro-inflammatory and antitumor immune environment in the synthetic MNs. For example, SASP signals such as CCL3, CXCL10, and CXCL11 (Fig. 4f) can direct CD8^+^ T cell recruitment^41, 42^ while XCL1 can recruit CD103^+^ cDC1 to promote immune control^43^. Pro-inflammatory signals such as IFNγ, TNFα, and IL12 (Fig. 4e) can enhance the cytotoxicity of NK cells and CD8^+^ T cells, allowing them to kill tumor cells through multiple mechanisms (e.g., IFNγ/TNFα cytokines, Fas/TRAIL ligands, and perforin granules, Fig. 4a)^44^. Signals that can induce cell senescence such as CXCL1, CXCL2, CXCL5, IFNγ, and TNFα (Fig. 4e, f)^45,46^ can arrest TC growth. Taken together, we concluded that signals from biomaterial-induced inflammation reversed cancer-induced suppression in the synthetic MNs and created an environment with potent antitumor immunity, contrasting with the lung environment dominated by immunosuppressive and pro-tumor signals (e.g., TGFβ, IL10, EGFR ligands) and immune cells with pro-tumor phenotypes (e.g., N2, M2, Fig. 4a–c).

### Synthetic MNs and lungs selectively recruit N1- or N2-polarized neutrophils through different chemoattractants in metastatic breast cancer

**Fig. 5:**
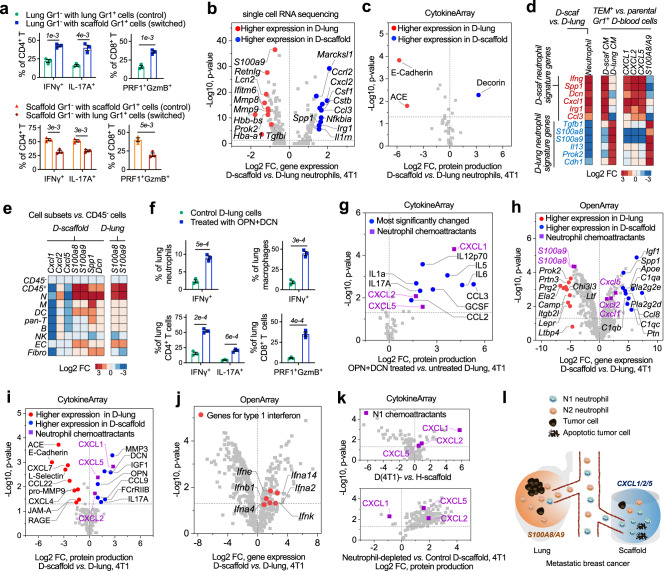
CXCL1, CXCL2, and CXCL5 recruit N1-polarized neutrophils to the dormant synthetic MNs, while S100A8/A9 recruits N2-polarized neutrophils to the immunosuppressive lungs in BALB/c mice with 4T1 metastatic breast cancer. **a** Phenotypic change of T cells in diseased scaffolds and lungs after lung- or scaffold-derived Gr1^−^ cells were separately incubated with niche-specific Gr1^+^ cells in vitro. **b**, **c** Differential gene expression and protein production in neutrophils derived from diseased scaffolds and lungs. **d** Signature genes for neutrophils derived from diseased scaffolds or lungs, and their expression in circulating neutrophils transmigrating towards conditioned media and neutrophil chemoattractants. TEM^+^ cells were isolated from Boyden chambers in vitro. **e** Expression of genes for neutrophil chemoattractants, osteopontin (OPN, *Spp1* gene), and decorin (DCN, *Dcn* gene) proteins in cell subsets derived from diseased scaffolds and lungs. N neutrophil, M macrophage, EC endothelial cell, Fibro fibroblast. **f**, **g** Immune cell phenotypes (**f**) and protein production (**g**) in diseased lung cells after incubating with osteopontin and decorin proteins in vitro. **h**, **i** Differential gene expression and protein production in diseased scaffold and lung cells. **j** Expression of genes for type 1 interferon in diseased scaffolds compared to diseased lungs. **k** N1 neutrophil chemoattractants secreted by diseased lungs compared to those secreted by healthy lungs (top panel), and these chemoattractants secreted by diseased lungs of 4T1-bearing mice receiving anti-Gr1 antibodies compared to those secreted by the lungs of tumor-bearing mice without receiving antibodies (lower panel). **l** Schematic illustration of the selective recruitment of N1 and N2 neutrophils to scaffolds and lungs, respectively, in animal models of metastatic breast cancer responding to distinct groups of chemoattractants. Data are shown as Mean ± SEM and *p* values are from student’s two-tailed *t* test. *n* = 3 for (**a**), (**f**). Source data are provided as a Source Data file.

Subsequently, we identified neutrophils, the largest immune cell population in D-scaffold and D-lung (Fig. 1a), were the major cell population responsible for regulating the overall antitumor immunity in MNs. An in vitro study that exchanged Gr1^+^ cells in D-scaffold and D-lung niches indicated that T cells in lung-derived Gr1^−^ cells were activated by scaffold-derived Gr1^+^ cells, reflected by increased IFNγ and IL-17A production in CD4^+^ T cells and enhanced perforin expression in CD8^+^ T cells (Fig. 5a). Conversely, lung-derived Gr1^+^ cells suppressed T cell activity in D-scaffold. The multifaceted functions of neutrophils resulted from the heterogeneity of this cell population in the cancer setting^19–21,47^.

Large-scale analyses of the gene expression and protein production in neutrophils derived from synthetic MNs and endogenous lungs of BALB/c mice bearing 4T1 tumor (Fig. 5b, c) identified the niche-specific gene signature for neutrophils, including a six-gene panel (*Ifng*, *Spp1, Dcn, Cxcl1, Irg1, Ccl3*) for scaffold neutrophils and another six-gene panel (*Tgfb1, S100a8, S100a9, Il13, Prok2, Cdh1*) for lung neutrophils (Fig. 5d). These signature genes are associated with Th1/Th2/Th17 responses and anti/pro-tumor immunity. Specifically, IFNγ and osteopontin (OPN, encoded by Spp*1* gene) are associated with Th1 and Th17 responses, respectively, while IL13 is a Th2-type cytokine^48, 49^. CCL3 and CXCL1 are pro-inflammatory cytokines and recruit tumor-killing cells, while S100A8/A9 is linked to metastasis^25,41,42^. Besides, E-cadherin (encoded by *Cdh1* gene), an extracellular matrix (ECM) protein that promoted breast cancer metastasis^50^, was abundant in the immunosuppressive lungs. In contrast, the distinguishing ECM proteins in D-scaffold was decorin (DCN, encoded by *Dcn* gene) (Fig. 5d), mainly produced by scaffold neutrophils (Fig. 5e), has been demonstrated with antitumor and anti-metastasis functions in multiple types of cancers^51^.

DCN and OPN proteins were involved in FBR^52,53^. Previous studies suggested that OPN promoted Th17 inflammation and accelerated the proliferation and invasion of TCs at the primary tumor site^49^, and DCN acted as a TGFβ and growth factor antagonist and suppressed tumor growth^51^. Here, we identified the immune-boosting activity of OPN and DCN proteins in an immunosuppressive environment. In vitro incubation of recombinant OPN and DCN proteins with D-lung-derived unsorted cells resulted in increased IFNγ expression in neutrophils and macrophages, increased IFNγ and IL-17A production in CD4^+^ T cells, and enhanced perforin expression in CD8^+^ T cells (Fig. 5f). In addition, OPN and DCN proteins triggered D-lung cells to secrete pro-inflammatory proteins capable of activating immune cells or recruiting those with antitumor phenotypes. Those proteins included but were not limited to IL-12, IL-17A, CCL3, CXCL1, CXCL2, and CXCL5 (Fig. 5g). These results indicated that DCN and OPN, two ECM proteins abundant in the diseased scaffolds, can reverse cancer-induced immunosuppression in the diseased lungs. Notably, similar changes were not found in D-lung-derived Gr1^−^ cells incubated with OPN and DCN proteins (Fig. S11), suggesting these ECM proteins modulated Gr1^+^ neutrophils to influence the overall antitumor immunity in the MNs.

The niche-specific phenotype of neutrophils in D-scaffold and D-lung was partly ascribed to the selective recruitment of N1-polarized (antitumor phenotype) or N2-polarized (pro-tumor phenotype) neutrophils by two distinct groups of chemoattractants. The most abundant neutrophil chemoattractant in D-lung was S100A8/A9, contrasting with CXCL1, CXCL2, and CXCL5 in D-scaffold (Fig. 5h, i) that were largely derived from non-immune cells except CXCL2 originating from neutrophils (Fig. 5e). We performed an in vitro study to mimic the extravasation of blood-derived neutrophils responding to different neutrophil chemoattractants. Gr1^+^ cells derived from diseased blood were placed on the plate insert of Boyden chambers and those transmigrating to different chemoattractants located in the lower chamber were collected and then their gene expression was compared to parental cells. Our results indicated that the neutrophils transmigrating towards CXCL1, CXCL2, CXCL5, or D-scaffold CM overexpressed the signature genes of D-scaffold neutrophils. In contrast, the circulating neutrophils transmigrating towards S100A8/A9 or D-lung CM shared a similar gene expression pattern to D-lung neutrophils (Fig. 5d). These results suggested that CXCL chemokines and S100A8/A9 can selectively recruit N1 and N2 neutrophils from the heterogeneous neutrophils in circulation to scaffolds and lungs, respectively, to establish niche-specific phenotype of neutrophils. On the other hand, the synthetic MNs were abundant with type 1 interferon (Fig. 5j), creating a permissive condition for in situ polarization of neutrophils towards N1 in vivo^54^. However, we did not observe a change in the phenotype of blood-derived Gr1^+^ cells after incubating with D-scaffold CM in vitro (Fig. S12).

Unlike S100A8/A9, the abundance of CXCL1, CXCL2, and CXCL5 chemokines in scaffolds neither increased following the infiltration of cancer-induced neutrophils in MNs after tumor inoculation nor decreased after systemic depletion of neutrophils in tumor-bearing mice (Fig. 5k). These results suggested that pro-inflammatory signals CXCL1, CXCL2, and CXCL5 in the synthetic MNs, resulting from biomaterial-induced inflammation (Fig. 4f)^39,40^, were unrelated to the cancer disease.

Collectively, we compared the immune environments in the dormant synthetic MNs (scaffold implants) and the diseased lungs that promoted developing metastatic tumors. Scaffolds and lungs were derived from the same host with 4T1 metastatic breast cancer. The synthetic MNs overproduced CXCL1, CXCL2, and CXCL5 during pro-inflammatory responses to biomaterials. These CXCL chemokines selectively recruited N1 neutrophils from the circulation, modulating other immune cells and activating the overall antitumor immunity in the synthetic MNs. Conversely, the endogenous lungs of mice with 4T1 cancer were dominated by S100A8/A9 signals and N2 neutrophils, creating an immunosuppressive environment favorable for metastatic outgrowth (Fig. 5l). These findings revealed how the neutrophils with phenotypic and functional plasticity play the anti- or pro-tumor roles in 4T1 metastatic breast cancer.

### N1 Neutrophils drive lung metastatic dormancy in non-metastatic breast cancer

We subsequently extended our study from the dormant synthetic MNs and the immunosuppressive lungs in mice with 4T1 metastatic breast cancer to the dormant lungs in mice with non-metastatic or weakly metastatic breast cancer, intending to connect N1 and N2 neutrophils to breast cancer metastatic outcomes (dormancy or outgrowth) in the lungs.

**Fig. 6:**
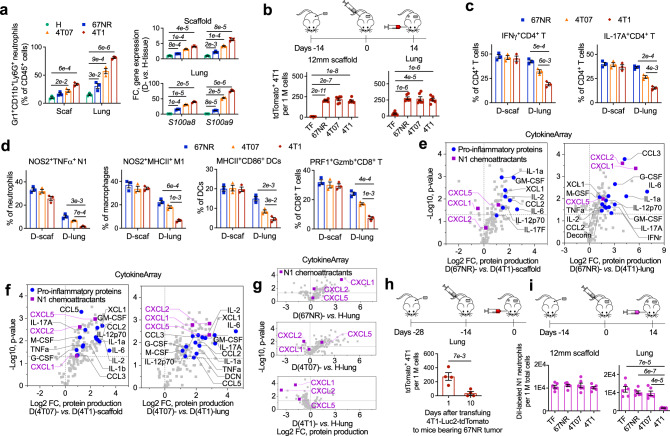
N1 neutrophils and their chemoattractants (CXCL1, CXCL2, and CXCL5) are abundant in the synthetic MNs and endogenous lungs of BALB/c mice with 67NR or 4T07 non-metastatic breast cancer. **a** The frequencies of Gr1^+^CD11b^+^Ly6G^+^ neutrophils and the expression of *S100a8* and *S100a9* genes in healthy tissues of tumor-free BALB/c mice and diseased tissues of mice bearing 67NR or 4T07 non-metastatic or 4T1 metastatic breast cancer tumor. Healthy tissues were the control. **b** In vivo extravasation of intracardially injected 4T1-Luc2-tdTomato cells to scaffolds and lungs of tumor-free mice and mice bearing non-fluorescent 67NR, 4T07, or 4T1 tumor. **c**, **d** The frequencies of IFNγ^+^ and IL-17A^+^ CD4^+^ T cells (**c**) and immune cells with pro-inflammatory and antitumor phenotypes (**d**) in diseased scaffolds and lungs of mice bearing 67NR, 4T07, or 4T1 tumors. Diseased tissues from 4T1-bearing mice were the control. **e**, **f** The proteins secreted by diseased tissues of mice bearing 67NR (**e**) or 4T07 (**f**) tumor compared to those secreted by diseased tissues of mice bearing 4T1 tumor. **g** The proteins secreted by diseased lungs of mice bearing 67NR, 4T07, or 4T1 tumor compared to those secreted by healthy lungs of tumor-free BALB/c mice. **h** 4T1-Luc2-tdTomato cells that were transfused to BALB/c mice bearing 67NR tumor through intracardiac injection were attracted to the lungs but failed to grow to a secondary tumor. **i** In vivo extravasation of intracardially injected N1 neutrophils to scaffolds and lungs of tumor-free BALB/c mice and mice bearing 67NR, 4T07, or 4T1 tumor. Gr1^+^ splenocytes subpopulation migrating towards CXCL1 in the Boyden chamber were isolated, and these neutrophils with verified N1 phenotypes were fluorescently labeled with DiI dye and then transfused into mice by intracardiac injection. Data are shown as Mean ± SEM and *p* values are from student’s two-tailed *t* test. *n* = 3 for (**a**), (**c**), (**d**), *n* = 6 for (**b**), (**i**), and *n* = 4 for (**h**). Source data are provided as a Source Data file.

67NR and 4T07 are two non-metastatic cell lines isogenic to metastatic 4T1 cells. 67NR cells failed to leave the primary site after orthotopic inoculation in BALB/c mice, and 4T07 cells failed to form macroscopic lung metastases after metastatic seeding^55^. Similar to 4T1, 67NR, and 4T07 tumors induced expansion of Gr1^+^CD11b^+^Ly6G^+^ neutrophils and overproduction of S100A8/A9 in scaffolds and lungs of BALB/c mice (Fig. 6a). Interestingly, their increase was positively related to the aggressiveness of the breast cancers, which were in the order: 4T1 (the most aggressive) > 4T07 > 67NR. For example, the frequency of Gr1^+^CD11b^+^Ly6G^+^ neutrophils (*vs*. total CD45^+^ immune cells) increased from 15.2% in the healthy lungs to 30.8%, 57.1%, and 81.0% in the lungs of mice bearing 67NR, 4T07, and 4T1 tumor, respectively, at day 14 after tumor inoculation (Fig. 6a). Consistently, the expression of *S100a8* gene in the lungs of 67NR, 4T07, and 4T1 cancers were 15.3-fold, 31.1-fold, and 39.5-fold, respectively, higher than that in the healthy lungs. Due to the increased Gr1^+^CD11b^+^Ly6G^+^ neutrophils and S100A8/A9 proteins, the lungs of BALB/c mice bearing 67NR, 4T07, or 4T1 tumor can actively recruit intracardially injected 4T1-Luc2-tdTomato metastatic breast cancer cells (Fig. 6b). These results suggested that failed distant metastasis in mice bearing 67NR tumor might be due to the lack of metastasis driver genes in 67NR TCs rather than the lack of signals in MNs to attract metastatic cells.

Although D-scaffold and D-lung in animal models with 67NR or 4T07 non-metastatic breast cancer were favorable for metastatic seeding, these MNs developed Th1/Th17 responses (Fig. 6c) and were infiltrated with N1 neutrophils and other immune cells with antitumor phenotypes (Fig. 6d). They were also abundant with N1 chemoattractants (CXCL1, CXCL2, and CXCL5) and proteins developing pro-inflammatory and antitumor immune responses (e.g., IFNγ, TNFα, IL12, IL17A/F, CCL3, XCL1, DCN, Fig. 6e, f). For example, the abundances of CXCL1, an N1 chemoattractant, in the media conditioned by the lungs derived from mice bearing 67NR and 4T07 tumors were 47.8-fold and 3.4-fold, respectively, higher than that in the media conditioned by the lungs derived from 4T1-bearing mice. Besides, the production of N1 chemoattractants in the lungs of BALB/c mice dramatically decreased after inoculation of 4T1 metastatic cancer cells but did not decrease after inoculation of 67NR or 4T07 non-metastatic cancer cells (Fig. 6g), suggesting the antitumor immunity in the lungs was influenced by the metastatic properties of the primary tumor. Because the lungs of mice with non-metastatic breast cancer developed potent antitumor immunity, we found 4T1 tumor cells, which were attracted to the lungs responding to S100A8/A9 after they were transfused into the mice through intracardiac injection, were gradually eradicated in these dormant lungs and failed to develop into secondary tumors (Fig. 6h).

In this part, we compared two types of dormant niches in breast cancer, the dormant lungs in animals with non-metastatic breast cancer and the synthetic MNs in animals with metastatic cancer. Although these two types of dormant niches in breast cancer were different, they had common features, including (1) potent Th1 and Th17 pro-inflammatory response, (2) abundant immune cells with pro-inflammatory and antitumor phenotypes such as N1 neutrophils, and (3) abundant chemoattractants for N1 neutrophils (CXCL1, CXCL2, and CXCL5). In another experiment, we demonstrated that the high frequencies of N1 neutrophils in the dormant MNs of breast cancer were associated with high abundances of N1 chemoattractants in vivo. We placed Gr1^+^ splenocytes derived from 4T1-bearing BALB/c mice in the Boyden chamber and isolated the subgroup of cells migrating towards CXCL1, which were verified with antitumor phenotypes. And then, we stained these N1 neutrophils with DiI dye and transfused them into tumor-free BALB/c mice and mice bearing non-fluorescent 67NR, 4T07, or 4T1 tumors through intracardiac injection. The abundance of DiI^+^ splenocytes in the scaffolds and lungs of these mice was measured by flow cytometry the following day after injection. Our results indicated that DiI-labeled N1 neutrophils preferentially accumulated at MNs that were abundant with CXCL1, CXCL2, and CXCL5 in vivo, including the lungs in tumor-free mice and mice bearing non-metastatic 67NR or 4T07 tumor and the scaffolds in all the mice regardless of their healthy or diseased conditions (Fig. 6i). Our data indicated that both the dormant synthetic MNs and dormant lungs in breast cancer had the capability of actively recruiting N1 neutrophils from the circulation to create potent local antitumor immunity to suppress metastatic tumor growth.

### The abundance of the chemoattractants for N1 neutrophils in the lungs is influenced by the host immunity

**Fig. 7:**
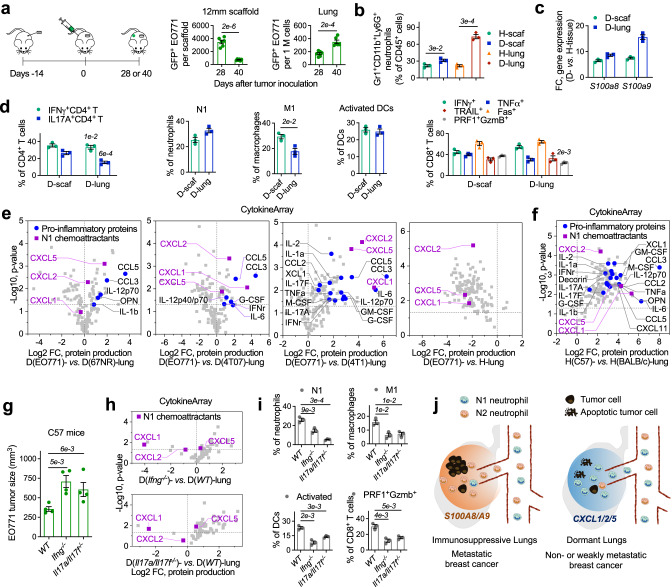
N1 neutrophils and their chemoattractants (CXCL1, CXCL2, and CXCL5) are abundant in the synthetic MNs and the endogenous lungs of C57 mice with EO771 weakly metastatic breast cancer. **a** EO771-GFP tumor cells migrated from the primary site to the diseased scaffolds and lungs but failed to form macroscopic metastases in C57 mice. The humane endpoint of C57 mice bearing EO771-GFP tumor without treatment was ~40 days after orthotopic tumor inoculation due to the large size of the primary tumor. **b**, **c** The frequencies of Gr1^+^CD11b^+^Ly6G^+^ neutrophils (**b**) and the expression of *S100a8* and *S100a9* genes (**c**) in diseased scaffolds and lungs of C57 mice bearing EO771-GFP tumor compared to healthy tissues of tumor-free C57 mice. **d** The frequencies of immune cells with pro-inflammatory and antitumor phenotypes in scaffolds and lungs derived from mice bearing EO771-GFP tumor. **e** The proteins secreted by diseased lungs of C57 mice bearing EO771 tumor compared to those secreted by diseased lungs of BALB/c mice bearing 67NR, 4T07, or 4T1 tumor or those secreted by healthy lungs of tumor-free C57 mice. **f** The proteins secreted by healthy lungs of tumor-free C57 mice compared to those secreted by healthy lungs of tumor-free BALB/c mice. **g** Size of the EO771-GFP primary tumor in wild type (WT), *Ifng*^*−/−*^, and *Il17a/Il17f*^*−/−*^ C57 mice at day 28 after orthotopic tumor inoculation. **h**, **i** The abundance of CXCL1, CXCL2, and CXCL5 proteins (**h**) and the frequencies of immune cells with pro-inflammatory and antitumor phenotypes (**i**) in the diseased lungs of wild type (WT), *Ifng*^−^, and *Il17a/Il17f*^−/−^ C57 mice bearing EO771-GFP tumor. **j** Schematic illustration of the selective recruitment of N1 and N2 neutrophils to the lungs of animal models of breast cancers with varying tumor aggressiveness responding to distinct groups of neutrophil chemoattractants, followed by creating an immunosuppressive or dormant environment in the lungs. Data are shown as Mean ± SEM and *p* values are from student’s two-tailed *t* test. *n* = 6 for (**a**), *n* = 3 for (**b**), (**c**), (**d**), (**i**), and *n* = 4 for (**g**). Source data are provided as a Source Data file.

In addition to the metastatic property of the primary tumor, we found that host immunity is another important factor influencing the abundance of N1 neutrophil chemoattractants in the MNs in breast cancer. EO771 is a weakly metastatic breast cancer^56^ and EO771-GFP TCs can seed scaffolds and lungs but fail to develop macroscopic metastases in immune-competent C57 mice orthotopically inoculated with EO771 tumor (Fig. 7a). Like 67NR and 4T07 tumors, EO771 tumor induced expansion of Gr1^+^CD11b^+^Ly6G^+^ neutrophils and overproduction of S100A8/A9 in the scaffolds and lungs of C57 mice (Fig. 7b, c). Meanwhile, potent antitumor immunity was developed in the lungs of C57 mice bearing EO771 tumor, characterized by high frequencies of N1 neutrophils and other immune cells with antitumor phenotypes (Fig. 7d) and high abundances of N1 chemoattractants and pro-inflammatory proteins (Fig. 7e). For example, the frequency of N1 neutrophils (*vs*. total neutrophils) was 32% in the lungs of C57 mice bearing EO771 tumor compared to 1.6% in the lungs of BALB/c mice bearing 4T1 tumor (Figs. 6d, 7d). The abundances of CXCL1, CXCL2, and CXCL5 in the lungs of C57 mice with EO771 tumor were 38.0-fold, 19.6-fold, and 10.8-fold, respectively, higher than that in the lungs of BALB/c mice with 4T1 cancer (Fig. 7e).

Compared to BALB/c mice bearing Th2-dominated host immunity, C57 mice have Th1 and Th17-skewed host immunity^57^. This difference resulted in a higher abundance of pro-inflammatory proteins and N1 neutrophil chemoattractants in the healthy lungs of tumor-free C57 mice compared to the healthy lungs of BALB/c mice (Fig. 7f). Specifically, the abundances of CXCL1, CXCL2, and CXCL5 in the healthy lungs of C57 mice were 39.7-fold, 4.9-fold, and 19.6-fold, respectively, higher than that in the healthy lungs of BALB/c mice. Although the EO771 tumor decreased the abundance of N1 chemoattractants in the lungs (Fig. 7e, D- *vs*. H-lung), due to the Th1 and Th17-skewed host immunity, diseased lungs of C57 mice bearing EO771 tumor had a higher abundance of pro-inflammatory proteins and N1 chemoattractants than the lungs of BALB/c mice bearing 67NR, 4T07, or 4T1 tumor (Fig. 7e).

When the host immunity of C57 mice was compromised by knocking down the *Ifng* gene or the *Il17*a/*Il17f* genes in the mice, the growth of EO771 tumor at the primary site accelerated (Fig. 7g). In mice with EO771 breast cancer, the abundances of CXCL1 in the diseased lungs of *Ifng*^−/−^ mice and *Il17a/Il17f*^−/−^ mice were 6% and 18% of that in the diseased lungs of mock C57 mice. The abundance of CXCL2 also decreased in the diseased lungs of gene-modified mice than that in the diseased lungs of mock mice while the abundance of CXCL5 did not decrease (Fig. 7h). Following the decrease of N1 chemoattractants, the frequency of N1 neutrophils (*vs*. CD45^+^ total immune cells) in the diseased lungs decreased from 26% in mock C57 mice to 14.6% in *Ifng*^−/−^ mice and 5.4% in *Il17a/Il17f*^−/−^ mice. Other immune cells with antitumor phenotypes also dramatically decreased in the diseased lungs of C57 mice bearing EO771 tumor after *Ifng* or *Il17a/Il17* genes were knocked out (Fig. 7i).

Through the study of multiple breast cancer models, we concluded that high abundances of N1 neutrophils and their chemoattractants (CXCL1, CXCL2, and CXCL5) were common and necessary to develop potent antitumor immunity and drive metastatic dormancy in the lungs that had been seeded with breast cancer TCs (Fig. 7j), and their abundance in the lungs was influenced by the metastatic properties of the primary tumor and the host immunity.

### The N1-to-N2 chemoattractant ratio is an indicator of lung metastatic dormancy, low tumor aggressiveness, and survival advantage in breast cancer

Our study in the animal models of breast cancer suggested that the extent of antitumor immunity in the MNs was largely dependent on the proportion of N1-to-N2 neutrophils. Dormant synthetic MNs included a large portion of N1 neutrophils and a small portion of N2 neutrophils while the neutrophils in the immunosuppressive lungs from the mice with 4T1 cancer were predominantly N2 and immature phenotypes (Fig. 4b). This N1-to-N2 proportion in the MNs can be reflected by the N1-to-N2 chemoattractant ratio, such as the *Cxcl1*-to-*S100a8* ratio. *Cxcl1* and *S100a8* have been suggested as prognostic genes associated with favorable and unfavorable clinical outcomes in breast cancer, respectively, in previous publications^58, 59^. Here, we hypothesized that the N1-to-N2 chemoattractant ratio, such as the *Cxcl1*-to-*S100a8* gene ratio, could be a better biomarker to predict the survival of breast cancer patients than the single gene since the ratio can indicate whether N1 is the dominant neutrophil phenotype in the MNs. Besides, this ratio might predict the risk of lung metastasis and the aggressiveness of primary tumors in breast cancer.

**Fig. 8:**
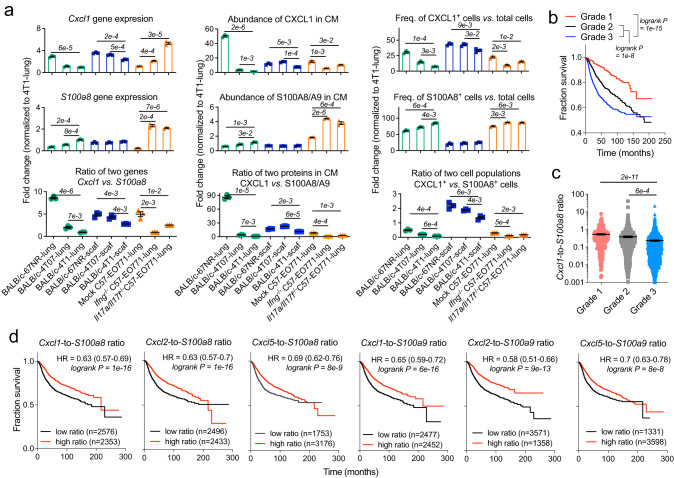
The N1-to-N2 chemoattractant ratio is a predictive biomarker of metastatic dormancy, low tumor aggressiveness, and survival advantage for breast cancer. **a** A positive correlation of the CXCL1-to-S100A8 ratio in MNs with the potential of metastatic dormancy in animal models of breast cancer. Scaffolds and lungs derived from animal models of different breast cancers were compared in terms of the expression of *Cxcl1* and *S100a8* genes in unsorted niche cells and their ratios (left panel), the abundance of CXCL1 and S100A8 proteins in the conditioned media and their ratios (middle panel), and the frequencies of CXCL1^+^ and S100A8^+^ cells in unsorted niche cells and their ratios (right panel). **b** Kaplan–Meier curves displaying the survival probability of patients with grade 1, 2, or 3 breast cancers. *p* values are from Mantel-Cox test. **c** The values of the *Cxcl1*-to-*S100a8* gene expression ratio in breast cancers with different grades. *p* values are from student’s two-tailed *t* test. **d** Kaplan–Meier curves displaying the survival probability of breast cancer patients with a low (black) or high (red) value of the expression ratio of genes for N1 chemoattractants (*Cxcl1* or *Cxcl2* or *Cxcl5*) to genes for N2 chemoattractants (*S100a8* or *S100a9*) at the primary tumor site. HR: hazard ratio; *n* = number of patients with available clinical data. *p* values are from Mantel-Cox test. Data for (**b**), (**c**), and (**d**) was retrieved from an online database^60^. Data are shown as Mean ± SEM. *n* = 3 for (**a**). In (**b**) and (**c**), *n* = 396 for grade 1, *n* = 1174 for grade 2, and *n* = 1286 for grade 3. Source data are provided as a Source Data file.

To verify this hypothesis, we first compared the values of the *Cxc1*-to-*S100a8* ratio in the synthetic MNs and lungs of multiple animal models of breast cancer with varying tumor aggressiveness and host immunity. We quantified the expression of two genes in unsorted niche cells (cells from the MNs), the abundances of two proteins in media conditioned by niche cells, and the frequencies of niche cells expressing CXCL1 or S100A8 proteins (Fig. 8a). Our results suggested that the *Cxcl1*-to-*S100a8* ratio was positively correlated with the potential of MNs to drive metastatic dormancy. For example, 4T1-lung (lungs of BALB/c mice bearing 4T1 tumor) had the lowest value and was also the only niche developing lethal metastases among all the MNs analyzed. Compared to 4T1-lung, lungs of BALB/c mice bearing non-metastatic 67NR or 4T07 tumor showed higher values of the *Cxcl1*-to-*S100a8* ratio, and the values suggested the likelihood of developing metastases was in the order of 4T1-lung > 4T07-lung > 67NR-lung, consistent with the activity of antitumor immunity in these MNs (Fig. 6d). In addition, a decrease in the value of the *Cxcl1*-to-*S100a8* ratio was observed in diseased lungs of *Ifng*^*−/−*^ and *Il17a/Il17f*^*−/−*^ transgenic C57 mice compared to the diseased lungs of mock C57 mice, indicating that Th1 and Th17 signals derived from the host immunity contributed to preventing lethal lung metastases in breast cancer.

Local pro-inflammatory signals induced by the immunogenicity of synthetic biomaterials activated the in situ antitumor immunity and drove metastatic dormancy in scaffolds regardless of cancer subtype. The values of the *Cxcl1*-to-*S100a8* ratio in the synthetic MNs derived from BALB/c mice bearing 67NR, 4T07, or 4T1 tumors were significantly higher than 4T1-lung (Fig. 8a). Notably, this ratio in the synthetic MNs and lungs concurrently decreased as an increase in the aggressiveness of breast cancer (67NR *vs*. 4T07 *vs*. 4T1) or as the disease progressed over time (days 7 *vs*. 14 *vs*. 21 in 4T1 cancer, Fig. S13). The similar trend of this ratio changes in the synthetic MNs and lungs suggested that scaffolds and lungs were simultaneously conditioned by cancer-induced systemic immune alterations.

Subsequently, we studied the clinical relevance of the N1-to-N2 chemoattractant ratio using an online database^60^ to analyze the association of this ratio with breast cancer grade (tumor aggressiveness) and breast cancer patients’ survival. This database includes transcriptomic data and multiple clinical characteristics (e.g., cancer grade) of breast cancer samples and patients’ survival data^60^. We compared the values of the *Cxcl1*-to-*S100a8* ratio in patients with grade 1, 2, or 3 breast cancers. Breast cancer with a high grade number is growing faster and more likely to spread than cancer with a low number, resulting in a poor prognosis (Fig. 8b). As expected, the *Cxcl1*-to-*S100a8* ratio had a significantly higher value in grade 1 and 2 breast cancers than in grade 3 cancers (Fig. 8c, grade 3 cancers are more aggressive than grade 1 and grade 2 cancers), like our observation in animal models that this ratio had a higher value in non-metastatic 67NR and 4T07 cancers than metastatic 4T1 cancer. Our data suggested that the N1-to-N2 chemoattractant ratio was well associated with the aggressiveness of breast cancer tumors.

Besides, Kaplan–Meier curves indicated that N1 chemoattractant genes (*Cxcl1*, *Cxcl2*, and *Cxcl5*) are favorable prognosis markers (HR < 1, HR: hazard ratio, *p* values ranging from 4e−3 to 1e−5) while N2 chemoattractant genes (*S100a8* and *S100a9*) are unfavorable prognosis markers (HR > 1, *p* values ranging from 4e−10 to 1e−12) for breast cancer (Fig. S14). And the N1-to-N2 chemoattractant ratios are favorable prognosis biomarkers associated with breast cancer patients’ survival (HR < 1, *p* values ranging from 8e−8 to 1e−16, Fig. 8d). Compared to single genes, the ratio of two genes (one favorable and one unfavorable) had a higher prognostic capacity for breast cancer. These results were consistent with our finding that the CXCL1/CXCL2/CXCL5 and S100A8/S100A9, which were chemoattractants for N1 and N2 neutrophils, respectively, played an antitumor or pro-tumor role in breast cancer metastasis, respectively.

In conclusion, the N1-to-N2 chemoattractant ratio in the primary tumor is a prognostic biomarker for breast cancer grade and patients’ survival. And the value of this ratio in the lungs is an indicator of breast cancer metastatic dormancy by revealing the abundance of N1 neutrophils and the activity of antitumor immunity.

## Discussion

Metastatic dissemination was suggested to rely on the interaction of “seeds” (TCs) and “soil” (MN environment) in Paget’s theory of cancer metastasis^2,3^. Dissemination of TCs is not random but occurs when TCs are attracted to specific pre-metastatic niches infiltrated with tumor-educated immune cells, resulting in metastatic organotropism^2^. Our study provided additional evidence to support and supplement this theory by verifying the contribution of organ-specific TC chemoattractants (e.g., S100A8/A9) to organ-tropic (e.g., lung-tropic) TC dissemination and proving that similar “soils” (S100A8/A9-abundant synthetic MNs and lungs) favor the metastatic seeding of similar “seeds” (TCs with lung metastasis gene signature). Our data also suggested that breast cancer and other types of cancer (e.g., B16F10, MC38, ID8) may employ a similar mechanism to promote lung-tropic metastatic dissemination as their subpopulations migrating towards S100A8/A9, a cytokine that predetermined lung metastasis in multiple cancers^25^, shared a similar gene expression pattern.

Similar “soil” in the synthetic MNs and the endogenous lungs may allow monitoring of cancer-induced alterations in the lungs by probing the immune changes in the subcutaneously implanted synthetic MNs. In a previous study^59^, we identified a 10-gene panel from the dynamics at the synthetic MNs throughout the process of 4T1 breast cancer progression. The changes of these genes in the scaffold implants aligned with their expression patterns in the lung biopsies and correlated with the disease progression^59^. A combination of the synthetic MNs and a scoring system based on this 10-gene panel successfully predicted the outcomes (survival or disease recurrence) in a post-surgery model of breast cancer metastasis^59^. Consistently, in the present study, we verified that the N1-to-N2 chemoattractant ratio, such as the *Cxcl1*-to-*S100a8* ratio, in the synthetic and endogenous MNs correlated with the aggressiveness of breast cancer and the disease progression. The concurrent immune changes in scaffolds and lungs suggested that implantable synthetic MNs can serve as a sentinel for metastatic conditioning of lungs to monitor cancer progression and metastasis. This strategy may be advantageous relative to lung biopsy, which is invasive and has risks such as blood clots and infection. The synthetic MNs can detect metastasis earlier than CT-based imaging that identifies macro-metastases. Early cancer detection and therapeutic intervention will prevent the MNs from deteriorating into a therapy-resistant environment that ultimately becomes lethal.

The synthetic MNs and endogenous lungs formed receptive environments for TC seeding because they were both infiltrated with cancer-induced neutrophils. However, neutrophils in two niches have opposing phenotypes and they activated and suppressed the antitumor immunity, respectively, resulting in distinct metastatic outcomes. These results revealed the multifaceted roles of neutrophils in regulating metastatic seeding, dormancy, and outgrowth of breast cancer cells^19–21,47^. Controversy surrounds neutrophil function in cancer as they show both pro- and antitumor functions. Previous studies suggested that circulating neutrophils in cancer patients include two morphologically distinct subpopulations, one with antitumor phenotype and the other with immunosuppressive phenotype^61^, but most studies focused on reporting the pro-tumor functions of neutrophils or myeloid cells in the cancer setting^19–21^. Our data addressed the controversy about the phenotypic and functional diversity of tumor-associated neutrophils by revealing that the circulating N1 or N2 neutrophils can be selectively recruited to MNs responding to two different groups of neutrophil chemoattractants. A small portion of cancer-induced S100A8/A9-producing neutrophils and a large portion of FBR-induced N1 neutrophils endow the synthetic MNs with the capability to recruit TCs from the circulation while suppressing their proliferation. More importantly, we found that as cancer progressed to the late stage, the in situ antitumor immunity in the synthetic MNs can still efficiently eliminate TCs, even though the frequency of N1 neutrophils decreased and the neutrophils in the scaffolds showed overall suppression of T cell proliferation in vitro.

Our data also underscored the importance of N1 neutrophils in driving breast cancer metastatic dormancy in the lungs, implying the potential therapeutic benefits of increasing N1 neutrophils and their chemoattractants in the endogenous lungs to activate antitumor immunity and prevent lethal lung metastases. This neutrophil-targeted anti-metastasis strategy might be more efficient and less toxic than the previous one that indistinguishably depletes Gr1^+^CD11b^+^ myeloid cells or Gr1^+^CD11b^+^Ly6G^+^ neutrophils regardless of their pro- or antitumor phenotypes through systematic administration of anti-Gr1 or anti-Ly6G antibodies^26^. In addition, we demonstrated that a high N1-to-N2 chemoattractant ratio is positively related to the survival advantage of breast cancer patients, and this ratio is influenced by the aggressiveness of breast cancer primary tumors and host immunity. This finding explains the diverse outcomes of breast cancer patients in clinic and provides insights into monitoring and driving metastatic dormancy in breast cancer.

In this study, we also demonstrated that the immunogenicity of synthetic biomaterials elicited potent antitumor immunity in the synthetic MN—a cargo-free porous PCL scaffold implant in tumor-bearing animal models. PCL scaffolds developed FBR featured by Th1/Th17 responses and cell senescence in tumor-free BALB/c mice^40^, and these FBR events were not changed in scaffolds of tumor-bearing BALB/c mice. Pro-inflammatory signals from FBR dominated cancer-induced suppressive signals and created an immune-active antitumor environment inhibiting TC proliferation. Previous studies suggested that myeloid cells played a crucial role in developing FBR induced by synthetic biomaterials^62^. Consistently, we found Gr1^+^CD11b^+^Ly6G^+^ granulocytic myeloid cells (neutrophils) activated Th1/Th17 responses and antitumor immunity in the PCL scaffolds of immune-competent BALB/c mice bearing breast cancer tumors. We also observed that in immune-compromised NSG mice lacking T and B cells and bearing defective macrophages, DCs, and NK cells, scaffold implants composed of PLG, another synthetic biomaterial degrading faster than PCL^22^, also developed in situ antitumor immunity and suppressed the proliferation of TCs in the metastatic breast cancer setting^12,13^.

Synthetic scaffolds in animal models of breast cancer captured and eradicated a portion of TCs from the circulation, reduced tumor burdens in the major organs, and provided a survival advantage compared to mice without scaffold implants^12,13^. These results implied the therapeutic potential of scaffold implants as a TC trap to combine with other adjuvant therapies to lessen metastasis^63,64^. Besides subcutaneous implants, we also found intravenous administration of PLG particles to BALB/c mice bearing 4T1 tumor suppressed lung metastasis compared to mice receiving PBS^65^. This benefit may result from the immune activation driven by PLG particles accumulated in the lungs after systemic administration. In addition to PCL and PLG, other synthetic biomaterials may also have immune-boosting activity in cancer by eliciting in situ pro-inflammatory myeloid immune responses^62^. Their capacity to activate antitumor immunity may depend on the biomaterial’s mechanical and physicochemical parameters that influence the immunogenicity of synthetic biomaterials in FBR, such as stiffness, surface charge, hydrophobicity, and chemical structure^66^.

Biological or natural materials (e.g., polypeptides, polysaccharides, and tissue ECM)^22^ were also recently identified with antitumor activity yet employing a mechanism different from synthetic materials to activate in situ antitumor immunity. The study indicated that biological scaffolds suppressed primary tumor growth through material-elicited Th2-associated pro-regenerative immune responses after biomaterials and TCs were co-injected into animal models^67,68^. Unlike synthetic materials that rely on neutrophils to activate antitumor immunity, biological materials require macrophage and CD4^+^ T cells to inhibit tumor growth. Therefore, they lost the tumor-suppressing ability in the gene-modified mice lacking adaptive immune systems^62,68^. Different mechanisms by which synthetic and biological materials boost antitumor immunity provide an opportunity to dissect the roles of multiple classes of immune cells in activating antitumor immunity. These engineered scaffolds, including the implantable MNs and the co-injection model, can also serve as in vivo niche-mimicking models to study cancer dormancy at the primary or metastatic site^69,70^. In addition, dormant synthetic MNs include assorted antitumor signals, which have the great potential to be developed to the next-generation immune-boosting anticancer drugs, such as osteopontin and decorin.

Overall, our study identified that porous scaffold implants made of synthetic biomaterials could act as dormant synthetic MNs in metastatic breast cancer, recruiting lung-tropic TCs from the circulation while suppressing their growth. Our study of this in vivo MN-mimicking model advanced our understanding of the mechanism underlying breast cancer lung metastasis by revealing how neutrophils with different phenotypes play roles in metastatic seeding, outgrowth, and dormancy of breast cancer cells in the lung metastasis. Based on these findings, a couple of proteins (e.g., N1 chemoattractants, osteopontin, and decorin) were identified with great immunotherapeutic potential to drive breast cancer metastatic dormancy, and a prediction model (N1-to-N2 chemoattractant ratio) was suggested to monitor the risk of metastatic outgrowth in the lungs and predict outcomes for breast cancer.

## Methods

### Chemicals and recombinant proteins

Poly(ε-caprolactone) (PCL, ester terminated) was from LACTEL. Matrigel and Transwell inserts were from Corning. VybrantTM multicolor cell-labeling kit, CellTracker Green CMFDA, and ACK lysis buffer were from Thermo Fisher Scientific. D-luciferin was from PerkinElmer. 4% paraformaldehyde (PFA), TruStain FcX PLUC (anti-mouse CD16/32), cell staining buffer, recombinant human TNFα (carrier free, #570102), recombinant mouse S100A8/A9 heterodimer (carrier-free, #765504) and recombinant mouse CCL2 (MCP-1) (carrier-free, #578402) were from BioLegend. Mouse osteopontin (#441OP050) and mouse decorin (#1060DE100) recombinant proteins were from R&D systems. Liberase TL, DNase I, and mouse CXCL12/SDF-1α (#SRP4388) were from Sigma. Cell dissociation buffer (enzyme-free) was from Gibco. All the other chemicals were from Sigma.

### Primers

Primers were from RealTimePrimers, including mouse cytokine primer library I (MCA-1), mouse cytokine primer library II (MCA-II), mouse tumor invasion/metastasis primer library (MTIM-1), mouse cytokine and chemokine receptor primer library (MCCR-1), human breast cancer signaling primer library (HTIM-1), and human invasion primer library (HTIM-1), and human cytokine and chemokine receptor primer library (HCCR-1).

### Antibodies

Alexa Fluor 488-labeled anti-mouse S100A8 antibody (clone 63N13G5, #27067, 1: 50 dilution) and Alexa Fluor 647-labeled anti-mouse CXCL1 monoclonal antibody (clone 1174A, #IC4532R, 1: 50 dilution) were from Novus Biologicals. PE-Cyanine7 NOS2 monoclonal antibody (CXNFT, #25-5920-80, 1:200 dilution) and PE-Cyanine7 Arginase 1 monoclonal antibody (#25-3697-80, 1:40 dilution) were from Thermo Fisher Scientific, eBioscience. Anti-CDKN2A/p16INK4a antibody was from AbCam (EPR20418, #ab211542, 1:200 dilution).

All the other antibodies used in flow cytometry were from BioLegend, including Pacific Blue™ anti-mouse Ly-6G/Ly-6C (Gr-1) antibody (RB6-8C5, #108429, 1:50 dilution), Pacific Blue™ anti-mouse CD19 antibody (6D5, #115526, 1:200 dilution), Brilliant Violet 421™ anti-mouse F4/80 antibody (BM8, #123131, 1:100 dilution), Brilliant Violet 421™ anti-mouse CD3 antibody (17A2, #100227, 1:100 dilution), Brilliant Violet 510™ anti-mouse/human CD11b antibody (M1/70, #101245, 1:20 dilution), Brilliant Violet 510™ anti-mouse CD4 antibody (GK1.5, #100449, 1:50 dilution), Brilliant Violet 510™ anti-mouse CD8a antibody (53-6.7, #100751, 1:50 dilution), Brilliant Violet 605™ anti-mouse CD11c antibody (N418, #117333, 1:100 dilution), Brilliant Violet 605™ anti-mouse IFN-γ antibody (XMG1.2, #505839, 1:20 dilution), FITC anti-mouse Ly-6C antibody (HK1.4, #128005, 1:200 dilution), FITC anti-mouse CD8a antibody (53-6.7, #100705, 1:50 dilution), Alexa Fluor® 488 anti-mouse CD206 (MMR) antibody (C068C2, #141709, 1:50 dilution), FITC anti-mouse I-A/I-E (MHC class II) antibody (M5/114.15.2, #107605, 1:200 dilution), FITC anti-mouse CD103 antibody (2E7, #121419, 1:50 dilution), Alexa Fluor® 488 anti-mouse/rat/human CD27 antibody (LG.3A10, #124221, 1:100 dilution), FITC anti-human/mouse Granzyme B antibody (GB11, #515403, 1:20 dilution), FITC anti-mouse TNFα antibody (MP6-XT22, #506303, 1:200 dilution), PE anti-mouse Ly-6G antibody (1A8, #127607, 1:100 dilution), PE anti-mouse IFN-γ antibody (XMG1.2, #505807, 1:100 dilution), PE anti-mouse CD253 (TRAIL) antibody (N2B2, #109305, 1:100 dilution), PE anti-mouse Perforin antibody (S16009A, #154305, 1:50 dilution), PE/Cyanine7 anti-mouse F4/80 Recombinant antibody (QA17A29, #157307, 1:25 dilution), PE/Cyanine7 anti-mouse CD49b antibody (HMα2, #103517, 1:200 dilution), PE/Cyanine7 anti-mouse IL-17A antibody (TC11-18H10.1, #506921, 1: 100 dilution), APC anti-mouse CD11c antibody (N418, #117309, 1:100 dilution), APC anti-mouse CD3 antibody (17A2, #100235, 1:50 dilution), APC anti-mouse CD95 (Fas) antibody (SA367H8, #152603, 1:200 dilution), APC anti-mouse CD86 antibody (GL-1, #105011, 1: 100 dilution), APC anti-mouse IL-10 antibody (JES5-16E3, #505009, 1:100 dilution), APC anti-mouse ESAM antibody (1G8/ESAM, #136207, 1:100 dilution), APC anti-mouse TCR γ/δ antibody (GL3, #118115, 1:100 dilution), and Alexa Fluor® 700 anti-mouse CD45 antibody (30-F11, #103127, 1:200 dilution).

### Cells

4T1 (non-fluorescent) metastatic murine breast cancer cells line (#CRL-2539) was from ATCC. Human umbilical vein endothelial cell (HUVEC, #C2519A) was from Lonza. 4T1-Luc2-tdTomato cell line (#: 125669) was from Caliper. 67NR and 4T07 non-metastatic murine breast cancer cell lines were from Karmanos Cancer Institute at Wayne State University. EO771-GFP murine breast cancer cell line was kindly provided by the lab of Gary and Kathryn Luker in the center for molecular imaging at the University of Michigan. B16F10 murine melanoma cell line, MC38 murine colon carcinoma cell line, and ID8 murine ovarian cancer cell line were kindly provided by the lab of Weiping Zou in the department of surgery at the University of Michigan.

### Animals

Female BALB/c mice (#000651, 6–7 weeks), female C57BL/6 mice (#000664, 6–7 weeks), female *Ifng*^−/−^ C57BL/6 mice (B6.129S7-*Ifng*^*tm1Ts*^, #002287, 6–7 weeks) mice and female *Il17a/Il17f*^−/−^ C57BL/6 mice (B6.Cg-*Il17a/Il17f*^*tm1.1lmpr*^*Thy1*^*a*^, #034140, 6–7 weeks) were from the Jackson Lab. Mice were housed in cages that were well ventilated, softly lit and subject to a light dark cycle. Mouse rooms and cages were kept at a temperature range of 20–24 °C with humidity at 40–60%.

All animal studies were performed in accordance with institutional guidelines and protocols (PRO00009715) approved by the University of Michigan Institutional Animal Care and Use Committee.

### Cell culture

4T1 and 4T1-Luc2-tdTomato cancer cells were cultured in RPMI-1640 medium (Gibco) supplemented with 10% fetal bovine serum (FBS, Gibco). HUVEC was cultured in EBM-2 (endothelial cell basal medium-2) supplemented with 10% FBS and EGM^TM^-2 SingleQuots (Lonza). 67NR and 4T07 cells were cultured in high glucose Dulbecco’s Modified Eagle’s Medium (DMEM, Gibco) with 10% FBS, 2 mM L-glutamine, and 1% (v/v) nonessential amino acids. EO771-GFP cancer cells were cultured in DMEM with 10% FBS and 20 mM HEPES. B16F10 and MC38 cancer cells were cultured in DMEM with 10% FBS. ID8 cancer cells were cultured in high glucose DMEM with 4% FBS, 5 µg/ml insulin, 5 µg/ml transferrin and 5 ng/ml sodium selenite. All the cells were incubated at 37 °C with 5% of CO_2_ and full humidify.

### Microporous PCL scaffold fabrication

Microporous poly(ε-caprolactone) (PCL) scaffolds were fabricated by salt leaching technique. First, PCL were prepared as described previously^12–14,59,65^. Next, PCL microspheres were mixed with sodium chloride particles (250-425 µm) at a 1:30 (w/w) ratio in a mixer (ROSS) for 30 min at 85 °C. Subsequently, 6.2 g of polymer/salt mixture was placed in a 40 mm diameter steel die and pressed at 1500 psi for 45 s in bench top manual presses (Carver) to form a polymer/salt disc (~2 mm in height). After leaching the salt with distilled water overnight, the polymer disc was dried with Kimwipes and frozen at −80 °C for at least 1 h. To prepare scaffolds with desired size for implantation, the polymer disc was placed on dry ice and cut by disposable biopsy punch (5 mm, 8 mm, or 12 mm in diameter, Miltex). Microporous scaffolds were disinfected by 70% ethanol, rinsed with sterile water, air dried on a sterile gauze pad, and then stored at −80 °C until the time of implantation.

### Subcutaneous scaffold implantation

Scaffolds were implanted to the dorsal subcutaneous space of female BALB/c mice or female C57BL6 mice (8–10 weeks old) as described previously^12–14,59,65^. Mice were implanted with a maximum of two 8 mm or 12 mm scaffolds or a maximum of eight 5 mm scaffolds.

### 4T1 metastatic breast cancer model with orthotopic inoculation

At two weeks after scaffold implantation, 2 × 10^6^ of non-fluorescent 4T1 or fluorescent 4T1-Luc2-tdTomato cancer cells in 50 µl of PBS were injected into the right mammary fat pad of female BALB/c mice (orthotopic inoculation). 4T1 cells spontaneously metastasized to lungs and other organs from the primary site. The humane endpoint for this cancer model was ~28 days after tumor inoculation due to the burden of breath caused by lung metastases.

### 67NR and 4T07 non-metastatic breast cancer model with orthotopic inoculation

At two weeks after scaffold implantation, 2 × 10^6^ of non-fluorescent 67NR or 4T07 cancer cells in 50 µl of PBS was injected into the right mammary fat pad of female BALB/c mice. 67NR and 4T07 breast cancer failed to develop macroscopic metastases in the lungs at day 28 after tumor inoculation.

### EO771 weakly metastatic breast cancer model with orthotopic inoculation

At two weeks after scaffold implantation, 2 × 10^6^ of fluorescent EO771-GFP cancer cells in 50 µl of PBS was injected into the right mammary fat pad of female C57BL/6 mice, including wild-type and gene-modified mice. EO771 cells spread to lungs but did not develop macroscopic metastases before the humane endpoint for this cancer model, which was at ~40 days after tumor inoculation when the size of the primary tumor exceeded 2 cm in any dimension.

For all the breast cancer models described above, animals were euthanized when the primary tumor reached 2 cm in any dimension, ulceration of over half the surface area of the tumor, or changes in behavior or body condition indicative of morbidity (increase/decrease in respiration, appearance, weight loss).

### In vivo extravasation of 4T1-Luc2-tdTomato cancer cells to scaffolds and lungs of BALB/c mice (experimental metastasis model)

At two weeks after subcutaneous implantation of scaffolds, BALB/c mice received orthotopic inoculation of 2 × 10^6^ of non-fluorescent 4T1, 67NR, or 4T07 cancer cells (tumor-bearing, diseased group, 3–6 mice per group) or PBS (tumor-free, healthy group). At two weeks after inoculation of 4T07 and 4T1 cells and around three weeks after inoculation of 67NR cells, all three types of tumors grew to around the same size. At this time point, 1 × 10^6^ of fluorescent 4T1-Luc2-tdTomato cells in 100 µl of PBS was injected into the left ventricle of the mouse through U-100 insulin syringes with 29 G needles (Excel Comfort Point). Metastatic 4T1-Luc2-tdTomato cells would extravasate to metastatic sites from the circulation^71^. At the second day after intracardiac injection, scaffolds and lungs were collected and prepared to single-cell suspensions, and then the abundance of tdTomato^+^ 4T1 cells in these tissues were measured by flow cytometer.

To study the extravasation activity of 4T1 cells at different stages of cancer, 4T1-Luc2-tdTomato cells were intracardially injected to BALB/c mice bearing non-fluorescent 4T1 tumor at days 7, 14, and 28 after orthotopic tumor inoculation, which represented early, intermediate, and late stages of cancer, respectively. And the abundance of tdTomato^+^ 4T1 cells at scaffolds and lungs was measured by flow cytometer the day following intracardiac injection.

### In vivo extravasation of N1-polarized splenocytic Gr1^+^ cell subpopulation to scaffolds and lungs of BALB/c mice

The diseased spleen was retrieved from 4T1-bearing BALB/c mice at two weeks after orthotopic tumor inoculation, and then prepared to single-cell suspensions. Splenocytic Gr1^+^ cell population was isolated by magnetic-activated cell sorting, and then 4 × 10^5^ cells in 1 ml of serum-free RPMI-1640 media were added to 6-well Transwell insert (24 mm in diameter, 8 µm pore size) that had been covered with a TNFα-treated HUVEC layer. The lower chamber in plates was then added with 2 ml of 40 ng/ml CXCL1. Splenocytic Gr1^+^ cells transmigrating towards CXCL1 into the lower chamber were collected the next day, and then stained with DiI dye derived from VybrantTM multicolor cell-labeling kit following manufacturer’s instructions.

1 × 10^6^ of DiI-labeled splenocytic Gr1^+^ cells in 100 µl of PBS was intracardially injected to the mouse. Scaffolds and lungs were collected the next day, prepared to single-cell suspensions, and then the abundances of DiI^+^ cells in these tissues were measured by flow cytometer. Four groups of BALB/c mice, including tumor-free mice and mice bearing non-fluorescent 4T1, 67NR, or 4T07 tumor, received intracardiac injection of DiI-labeled splenocytic Gr1^+^ cells at two weeks after orthotopic tumor injection.

### In vitro extravasation of cancer cells

The activity of in vitro extravasation of cancer cells towards chemoattractants or conditioned media was studied in the Boyden chamber model under static conditions. Transwell inserts (6.5 mm in diameter, 8 µm pore size, Corning) were incubated with 10% Matrigel in ice-cold PBS at 4 °C overnight to allow gel coating. Matrigel was removed the next day, and then 4 × 10^4^ HUVEC cells in 0.5 ml of complete cell media were seeded into the 24-well Transwell insert and incubated at 37 °C for two days to allow the formation of a confluent HUVEC monolayer on the upper membrane surface of the insert. The HUVEC monolayer was activated by incubating with 20 ng/ml TNFα for 4 h at 37 °C, followed by rinse with PBS for three times.

Next, cancer cells growing in flasks were collected and stained with CellTracker Green CMFDA dye following the manufacturer’s instructions. 4 × 10^4^ CMFDA-labeled cancer cells in 100 µl of serum-free media were added to the Transwell insert covered with HUVEC monolayer (top chamber), and then 600 µl of conditioned media prepared by unsorted niche cells or their subsets, or 20 ng/ml S100A8/A9 chemoattractant (positive control), or serum-free media (negative control) was added to the reservoir plate (lower chamber). Two days later, the non-transmigrated cancer cells (TEM^-^ cells) on the upper membrane surface were removed by a PBS-soaked cotton swab, and then transmigrated cancer cells (TEM^+^ cells) on the reservoir side of the insert were fixed by 4% PFA, imaged by a fluorescence microscope (Zeiss) and analyzed with ImageJ software.

To neutralize S100A8/A9 chemoattractants, conditioned media or 20 ng/ml S100A8/A9 in serum-free media (positive control) were pre-incubated with 5 µg/ml anti-S100A8/A9 antibody at 37 °C for 1 h prior to placing to the lower chamber.

### Preparation of single-cell suspension

Fresh tissues were cut into 1–2 mm pieces in the digestion buffer (serum-free RPMI-1640 with 0.1WU/ml liberase TL and 150 U/ml DNase I), and then incubated at 37 °C for 30 min. Cells and the dissociated tissues were passed through 70 µm strainers to obtain single-cell suspensions. Red blood cells were removed by ACK lysis buffer following the manufacturer’s instructions.

### Isolation of immune cell subsets from diseased tissues

Cell subsets were isolated by magnetic-activated cell sorting (MACS) using kits and microbreads purchased from Miltenyi Biotec following the manufacturer’s instructions. Kits and microbeads used to isolate different cell subsets are listed in Table S1.

### Isolation of MDA-MB-231BR metastatic cancer cells from orthotopic cancer model

NSG mice received orthotopic inoculation of MDA-MB-231BR (231BR) human breast cancer cells. Metastatic cancer cells were isolated from lungs and scaffold implants and expanded in vitro as described before^14^.

### Isolation of cancer cell subpopulations transmigrating towards chemoattractants or conditioned media from Boyden chamber

In the Boyden chamber, 4 × 10^5^ HUVECs in 1 ml of complete media were seeded into the 6-well Transwell inserts (24.5 mm in diameter, 8 µm pore size, Corning) and incubated at 37 °C for two days to allow formation of a confluent monolayer on the top of the porous filter membrane. The HUVEC monolayer was activated by 20 ng/ml TNFα for 4 h, and then rinsed with PBS three times. Subsequently, 4 × 10^6^ cancer cells in 1 ml serum-free media were added to the insert (top chamber), and then 2 ml of conditioned media, or 20 ng/ml S100A8/A9, or 100 ng/ml CXCL12, or 40 ng/ml CCL2, or 0.1% FBS were added to the reservoir plate (lower chamber). Cancer cell chemoattractants were diluted in serum-free media. Two days later, transmigrated (TEM^+^) cancer cells were detached by immersing the reservoir side of the insert in cell dissociation buffer (enzyme-free) for 1 h at 37 °C and then collected.

### Isolation of circulating or splenocytic Gr1^+^ cell subpopulations transmigrating towards neutrophil chemoattractants or conditioned media from Boyden chamber

Diseased blood and spleen were retrieved from 4T1-bearing BALB/c mice two weeks after orthotopic tumor inoculation and prepared to single-cell suspensions. Red blood cells were removed by ACK lysis buffer following manufacturer’s instructions and then Gr1^+^ cells were isolated by magnetic-activated cell sorting. Subsequently, 4 × 10^6^ blood-derived (circulating) or spleen-derived (splenocytic) Gr1^+^ cells suspended in 1 ml serum-free RPMI-1640 media were added to the 6-well Transwell insert (top chamber) that had been covered with a TNFα-activated HUVEC monolayer. And then 2 ml of conditioned media or 40 ng/ml of CXCL1, CXCL2, CXCL5 or S100A8/A9 were added to the reservoir plate (lower chamber). Neutrophil chemoattractants were diluted in serum-free media. Circulating or splenocytic Gr1^+^ cells transmigrating to the lower chamber were collected the following day.

### Preparation of condition media

Tissues were derived from tumor-free mice or tumor-bearing mice at two weeks after orthotopic tumor inoculation and then prepared to single-cell suspensions. Red blood cells were removed by ACK lysis buffer.

To prepare conditioned media, unsorted cells or cell subsets isolated by magnetic-activated cell sorting were washed with PBS three times, suspended in serum-free media (phenol red-free RPMI-1640 medium) at a concentration of ~5 × 10^6^ cells/ml, and incubated at 37 °C for 24 h. After incubation, cell pellets were removed by centrifugation, and the conditioned media were stored at −80 °C. Total protein concentration of the conditioned media was quantified by BCA protein assay kit (Thermo Fisher Scientific), and then the conditioned media were diluted to 0.3 mg/ml for in vitro studies.

### Identification of the abundance of fluorescent cancer cells or neutrophils in scaffolds and lungs with flow cytometry

The abundance of tdTomato^+^ 4T1 cells, DiI^+^ splenocytic Gr1^+^ cells, and GFP^+^ EO771 cells in the single-cell suspensions of scaffolds and lungs were quantified by flow cytometer (BioRad, ZE5) with Everest software at the University of Michigan Flow Cytometry core, and then analyzed with FlowJo_V10. > 100,000 cells were collected and analyzed.

### Analysis of the frequency of immune cells in scaffolds and lungs with flow cytometry

Scaffolds and lungs were retrieved two weeks after orthotopic tumor inoculation and then prepared to single-cell suspensions. Cells were blocked by anti-mouse CD16/32 antibody (1 µl/1 M cells/100 µl cell staining buffer) for 10 min on ice, and then stained by antibody cocktails on ice in the dark for 30 min for cell surface staining. Cells were washed with cell staining buffer and then fixed by 4% PFA. Cells were washed and then suspended in cell staining buffer the next day for flow cytometic analysis. More than 10,000 cells were collected and analyzed. Antibody cocktails for analyzing the frequencies of different classes of immune cells in the scaffolds and lungs are shown in Table S2.

### Analysis of the phenotype of immune cell subsets in scaffolds and lungs with flow cytometry

Cell surface staining was performed as described above. For cells requiring intracellular staining, cells were fixed and permeabilized using reagents in the BD Cytofix/Cytoper^TM^ fixations/permeabilization kit following the manufacturer’s instructions. Cells were then washed twice with permeabilization wash buffer and then incubated with antibody cocktails in the dark at 4 °C overnight. Cells were washed and then suspended in cell staining buffer the next day for flow cytometic analysis. More than 10,000 cells were collected and analyzed. Antibody cocktails for analyzing phenotype of immune cell subsets are in Table S3.

### Analysis of the frequency of CXCL1^+^ or S100A8^+^ cells in metastatic niches with flow cytometry

Scaffolds and lungs were retrieved two weeks after orthotopic tumor inoculation and prepared to single-cell suspensions. Cells were first blocked and stained with antibody cocktail for cell surface markers for 30 min on ice. Next, cells were fixed and permeabilized, and then stained with CXCL1 and S100A8 antibodies at recommended concentrations at 4 °C overnight. Cells were washed the next day and analyzed by flow cytometer. More than 10,000 cells were collected and analyzed. Antibodies are shown in Table S4.

### Identification of the abundance of 4T1-Luc2-tdTomato cells in scaffolds and lungs with in vivo bioluminescence imaging

Mice were intraperitoneally injected with 150 µl of 63 mM D-luciferin solution. 15 min later, bioluminescence imaging was conducted with an IVIS (PerkinElmer) to observe the bioluminescence intensity of 4T1-Luc2-tdTomato in scaffolds, lungs, or primary tumor. In this study, bioluminescence imaging was used to (1) measure the abundance of intracardially injected 4T1-Luc2-tdTomato cells in the scaffolds and lungs of mice bearing non-fluorescent 4T1 tumor; and (2) examine whether the 4T1-Luc2-tdTomato primary tumor was completely resected and then quantify lung metastases immediately following surgery.

### Quantitative RT-PCR (qRT-PCR)

Single-cell suspensions of unsorted niche cells or cell subsets were immediately used for RNA extraction. Total RNA was extracted using PureLink RNA mini kit (Thermo Fisher Scientific) and cDNA was then synthesized using iScript^TM^ cDNA synthesis kit (Bio-Rad) following manufacturers’ instructions. qRT-PCR was performed in 96-well plates on CFX Connect Real-Time System (Bio-Rad) with the following program: (1) 1 cycle of 95 °C for 10 min; (2) 40 cycles of 95 °C for 10 s and 58 °C for 45 s. A total of 10 µl of reaction mixture was measured in each well, which included 5 µl of 2 × SYBR Green supermix (Bio-Rad), 2 µl of 2 M primer mixture, 2 µl of cDNA (50 ng/ml) and 1 µl Nuclease-free water. Three replicates were prepared for each sample. Data analysis was conducted using the ΔΔC_t_ method in which all C_t_ values in each sample were first normalized to GAPDH. Each experiment was repeated at least three times.

### Open array

RNA isolation, cDNA synthesis, and Open array analysis were performed as described before^59,72^. Scaffold and lung tissues were retrieved from tumor-free BALB/c mice or 4T1-bearing BALB/c mice at two weeks after orthotopic tumor inoculation. Tissues were then flash-frozen by submerging them in isopentane on dry ice and homogenized in TRIZol reagent (Thermo Fisher Scientific). Total RNA from tissues was isolated with Directzol RNA Miniprep kit (Zymo Research) following the manufacturer’s instructions. Samples to be used for Open array analysis were assessed for RNA integrity with an RNA fragment analysis. And then cDNA was synthesized with the SuperScript^TM^ VILO^TM^ cDNA synthesis kit (Thermo Fisher Scientific) following the manufacturer’s instructions. High-throughput gene expression analysis of healthy and diseased tissues using OpenArray TaqMan^TM^ OpenArray^TM^ mouse inflammation panels (Thermo Fisher Scientific, QuantStudio^TM^ 12 K Flex) was performed at the University of Michigan DNA Sequencing Core. Two replicates were prepared for each sample.

### Single-cell RNA sequencing

Single-cell RNA sequencing (scRNA-seq) was used to analyze healthy and diseased tissues following our previous publications^59,73^. Briefly, scaffolds and lungs were collected from healthy BALB/c mice (*n* = 3) and 4T1-bearing mice two weeks after tumor inoculation (*n* = 3). Single-cell suspensions were isolated using a combination of enzymatic and mechanical dissociation. Cells were suspended in 0.01% bovine serum albumin at a concentration of 10^5^ cells per mL. Library preparation was completed with Drop-Seq followed by conversion to cDNA, expansion through PCR, and tagmentation^74^. Samples were sequenced using the Illumina HiSeq 4000 sequencer at the University of Michigan Advanced Genomics Core and aligned to the mm10 mouse genome. Cell identities, phenotypes, and differential gene expression were determined using the Seurat pipeline (version 3) in R^75^. scRNA-seq data can be accessed from ArrayExpress repositories (Accession: E-MTAB-8503).

### Expression levels of genes mediating organ-tropic metastasis in cancer cell subpopulations preferentially homing to different metastatic niches

Genes that mediate breast cancer metastasis to lungs and bones^28,30^ were used as a metric of induced organotropism and differential gene expression was measured to compare subpopulations attracted to metastatic niches relative to their parental cells. In vivo, MDA-MB-231BR cells at the primary site and those colonizing scaffolds and lungs of tumor-bearing NSG mice were isolated and expanded as described before^14^. In vitro, 4T1, B16F10, MC38, and ID8 cancer cell subpopulations transmigrating towards cancer cell chemoattractants or conditioned media in the Boyden chamber were isolated.

### Expression levels of signature genes for scaffold- or lung-derived neutrophils in circulating Gr1^+^ cells preferentially homing to different metastatic niches

A comparative study of Gr1^+^CD11b^+^Ly6G^+^ neutrophils derived from diseased scaffolds and lungs of 4T1-tumor bearing through single-cell RNA sequencing and cytokine array analyses identified a 6-gene panel (*Ifng*, *Spp1, Dcn, Cxcl1, Irg1, Ccl3*) as the signature genes of scaffold-derived neutrophils and a 6-gene panel (*Tgfb1, S100a8, S100a9, Il13, Prok2, Cdh1*) as the signature genes of lung-derived neutrophils.

Diseased blood or spleen were retrieved from 4T1-bearing mice at two weeks after orthotopic tumor inoculation, prepared to single-cell suspensions and then their Gr1^+^ cell populations were isolated by magnetic-activated cell sorting. Blood- or spleen-derived Gr1^+^ cell subpopulations transmigrating towards neutrophil chemoattractants or conditioned media were isolated from the Boyden chamber, and the expression of signature genes for scaffold- or lung-specific neutrophils in these Gr1^+^ cell subpopulations were compared with their parental Gr1^+^ cells.

### Expression levels of stemness marker genes in cancer cell seeding different metastatic niches

Expression of stemness marker genes^76^ was measured to compare cancer cells colonizing different metastatic niches relative to their parental cells derived from the primary tumor site. MDA-MB-231BR cells at the primary site and those colonizing scaffolds and lungs of tumor-bearing NSG mice were isolated and expanded as described before^14^.

### Analysis of transcription factor activity

Large-scale dynamic activity of transcription factors (TFs) in non-fluorescent 4T1 cells co-cultured with cells derived from diseased scaffolds and lungs of 4T1-bearing BALB/c mice was measured using TRanscriptional Activity CEll aRray (TRACER) as described in our previous study^31^. The principle of TRACER array is that cells infected with TF-reporting lentiviruses express TF reporters, which consist of a specific TF response element (TRE) cloned upstream of a minimal thymidine kinase promoter driving the gene for firefly luciferase (Fluc). When TFs are activated responding to different conditions, TFs bind to TRE, resulting in increased luciferase production and a proportional increase in luminescence when an excess of substrate is added during imaging. TF-reporting lentiviruses were prepared as described before and stored at −80 °C^31^.

In our study, we first seeded non-fluorescent 4T1 cells into 24-well plates at a density of 5000 cells per 500 µl per well and incubated them at 37 °C overnight to allow cell attachment. Media was replaced to fresh complete media including 10 MOI of each TF activity-reporting lentivirus the next day, and 4T1 cells were continued to be incubated at 37 °C for another two days to allow infection. In addition to 4T1 infected with TFs of interest, we also prepared 4T1 cells infected with TA Fluc and 4T1 cells without transfection, which were positive controls and negative controls, respectively, for data analysis.

Subsequently, 4T1 cells transfected with different TF reporters were collected and seeded into 96-well plates at a density of 1000 cells per 200 µl per well in complete media supplemented with 0.5 mM of D-luciferin. The next day, diseased scaffolds and lungs were retrieved from 4T1-bearing mice at two weeks after orthotopic tumor inoculation and prepared to single-cell suspensions. Diseased scaffold or lung cells were suspended in the complete media supplemented with D-luciferin, and then placed to 96-well plates at a ratio of cancer cells: niche cells = 1:16. Luminescence of TF reporters was measured by IVIS at 7 h and 24 h after 4T1 cells were co-cultured with niche cells. Data was analyzed as described before^31^. Three groups of cancer cells were studied, including 4T1 co-cultured with diseased scaffold cells, 4T1 co-cultured with diseased lung cells, and 4T1 without co-incubation with niche cells (control). Three replicates were prepared for each condition and TRACER experiments were repeated three times.

### ELISA

S100A8/A9 production in conditioned media was quantified by DuoSet^TM^ mouse S100A8/S100A9 heterodimer ELISA kit following manufacturers’ instructions. The abundance of IFNγ, TNFα, IL-12p40, TGFβ, CXCL1, CXCL2, and CCL3 in conditioned media was measured at the Immunology Core of the University of Michigan. The concentrations of cytokines or chemokines were normalized to the concentration of total proteins in the conditioned media that were quantified by BCA protein assay kit (Thermo Fisher Scientific). Three replicates were prepared for each sample and ELISA experiment was repeated three times.

### Cytokine array

Soluble proteins in conditioned media were detected by mouse CytokineArray C3 or C2000 (Ray Biotech Inc.) or mouse cytokine antibody array ab169813 (Abcam) following manufacturer’s instructions and imaged by ChemiDoc^TM^ Touch Imaging System (BioRad) and analyzed by Image Lab 6.1.

### Histological analysis of in situ expression of p16 protein in diseased tissues by immunohistochemistry

Diseased scaffolds and lungs were retrieved from 4T1-bearing BALB/c mice at two weeks after orthotopic tumor inoculation. Tissues were flash frozen in isopentane on ice, and then embedded in Tissue Tek O.C.T. Compound (Sakura Finetek). Frozen tissues were cryosectioned transversely into 12 µm thick sections and placed on Superfrost Plus microscope slides (Fisher). Tissues sections were then fixed by 4% PFA, washed, and permeabilized by 0.5% Triton in Tris buffer. To decrease the autofluorescence background, tissue sections were incubated with 0.3% (w/v) Sudan black in 70% EtOH overnight at room temperature, and then washed three times with 0.5% Triton. Subsequently, tissue sections were blocked in 10% normal goat serum in 0.1% Triton for 1 h at room temperature, washed, and then incubated with recombinant anti-CDKN2A/p16INK4a primary antibody [EPR20418] (AbCam, #ab211542, 1:200 dilution) in 0.1% Triton overnight at 4 °C. Tissue sections were stained with fluorescent secondary antibodies using Alexa Fluor^TM^ 488 Tyramide signal SuperBoost^TM^ kit (Invitrogen) and mounted in DAPI Fluoromount-G (SouthernBiotech), before imaging (Zeiss Axio Observer).

### In vivo fate of 4T1-Luc2-tdTomato cells in scaffolds and lungs in spontaneous metastasis models

BALB/c mice bearing two 12 mm PCL scaffolds received orthotopic inoculation of 4T1-Luc2-tdTomato cancer cells. And the abundance of fluorescent tdTomato^+^ 4T1 cells in the scaffolds and lungs were studied by flow cytometer at 7, 14, 21, and 28 days after tumor inoculation, which represented early, intermediate, intermediate-late, and late stages of cancer.

### In vivo fate of 4T1-Luc2-tdTomato cells in scaffolds and lungs in experimental metastasis models

BALB/c mice bearing two 12 mm PCL scaffolds received orthotopic inoculation of non-fluorescent 4T1 or 67NR cancer cells, and then received intracardiac injection of 1 × 10^6^ of 4T1-Luc2-tdTomato cancer cells in 100 µl PBS at two weeks after orthotopic tumor inoculation. The abundance of fluorescent tdTomato^+^ 4T1 cells in scaffolds and lungs were studied by flow cytometer at day 1 and day 12 (or day 10) after intracardiac injection.

### In vitro viability of 4T1-Luc2-tdTomato cancer cells after co-culturing with cells derived from D-lung or D-scaffold

4T1-Luc2-tdTomato cancer cells were seeded into 96-well plate at a density of 800 cells per 200 µl per well in complete media supplemented with 0.6 mM D-luciferin, and then incubated at 37 °C overnight to allow attachment. The next day, scaffolds and lungs were retrieved from 4T1-bearing BALB/c mice at two weeks after tumor inoculation and prepared to single-cell suspensions. Scaffold or lung cells were added to 4T1-Luc2-tdTomato cell cultures in D-luciferin-included complete media at the desired cancer cell to niche cell ratios, and then co-cultured for two days at 37 °C. The number of viable cancer cells was quantified by measuring the bioluminescence of 4T1-Luc2-tdTomato cancer cells using a plate reader (Synergy H1 hybrid multi-mode reader, BioTek).

### In vivo systemic depletion of Gr1^+^ cells in BALB/c mice bearing 4T1 tumor

BALB/c mice bearing two 12 mm PCL scaffolds received orthotopic inoculation of non-fluorescent 4T1 cancer cells. At day 7 and day 10 after tumor inoculation, 100 µg of InVivoMAb anti-mouse Ly6G/Ly6C (Gr1) antibodies (BioXCell, RB6-8C5, #BE0075) in 100 µl PBS was administrated to the treatment group (*n* = 4) by intraperitoneal injection while 100 µg of isotype control antibodies (BioXCell, LTF-2, #BE0090) was administrated to the control group (*n* = 4). 1 × 10^6^ of 4T1-Luc2-tdTomato cells in 100 µl were intracardially injected to both groups at day 12 after tumor inoculation. The next day, the abundance of 4T1-Luc2-tdTomato cells in the scaffolds and lungs were first measured through whole-body imaging of living mice by IVIS. Tissues were then harvested, prepared to single-cell suspensions, and the abundance of tdTomato^+^ cells in different tissues was quantified by flow cytometer.

### Metastases and survival rate of BALB/c mice after tumor resection surgery

Two groups of female BABL/c mice received orthotopic inoculation of 4T1-Luc2-tdTomato cancer cells. One group (*n* = 24) was implanted with two 12 mm PCL scaffolds in each mouse and the other group (*n* = 24) received mock surgery. At day 10, day 15, or day 20 after tumor inoculation, eight of scaffold-bearing mice and eight of scaffold-free mice received surgery to remove the primary tumor as described before^12,13^. Bioluminescence of 4T1-Luc2-tdTomato cells in the lungs were imaged by IVIS right after the surgery. And the survival rates of the scaffold-bearing and scaffold-free mice were monitored after the surgery.

### In vitro analysis of the suppressive function of scaffold- or lung-derived Gr1^+^ cells on T cell proliferation

Scaffolds, lungs, and spleen were retrieved from 4T1-bearing BALB/c mice at day 14 or day 21 after orthotopic inoculation of tumor cells and prepared into single-cell suspensions. Gr1^+^ cell populations were isolated from diseased scaffolds or lungs by magnetic-activated cell sorting. Naïve CD4^+^ T cells were isolated from diseased spleen by magnetic-activated cell sorting and stained using the CellTrace^TM^ Far Red cell proliferation kit (Thermo Fisher Scientific) following the manufacturer’s instructions. Next, T cells and Gr1^+^ cells were suspended in T cell media (glutamate-containing RPMI media supplemented with 10% FBS, 1% pen/strep, 1% nonessential amino acids, and 1% sodium pyruvate), and then seeded into 96-well plates previously coated with anti-CD3 antibodies (2.5 µg/ml, BioLegend,  145-2C11, #100302) at 37 °C for a minimum of 2 h.

Four groups of cultured T cells were prepared, including (1) unstimulated T cells that were not co-cultured with Gr1^+^ cells, representing the negative control group; (2) T cells that were stimulated to proliferate with the addition of a T cell stimulation cocktail (60 µg/ml anti-CD28 in T cell media, BioLegend, 37.51, #102102), which were not co-cultured with Gr1^+^ cells, representing the positive control group; (3) T cells that were stimulated to proliferate and co-cultured with lung-derived Gr1^+^ cells; and (4) stimulated T cells co-cultured with scaffold-derived Gr1^+^ cells. Co-cultures were plated at a ratio of T cells: Gr1^+^ cells = 1:5. Each group had three replicates and all groups were incubated at 37 °C for three days. Following the three-day incubation, cells were collected, stained with FITC-labeled anti-CD4 antibody (BioLegend, H129.19, #130308, 1:200 dilution), PE-labeled anti-CD25 antibody (BioLegend, 3C7, #101903, 1:50 dilution), and DAPI; and then T cell proliferation was measured by flow cytometer. CD4^+^ T cells were gated and their proliferation analyzed based on their Far Red signal.

### In vitro co-culture of D-scaffold- or D-lung-derived Gr1^−^ cells with Gr1^+^ cells derived from different metastatic niches

Diseased scaffolds and lungs were retrieved from 4T1-bearing BALB/c mice two weeks after tumor inoculation and prepared to single-cell suspensions. Gr1^+^ and Gr1^−^ cell subsets in diseased scaffolds and lungs were then separated by magnetic-activated cell sorting. Scaffold-derived Gr1^−^ cells were incubated with scaffold-derived Gr1^+^ cells (control) or lung-derived Gr1^+^ cells (switched) in the complete culture media at a ratio of Gr1^−^ cells: Gr1^+^ cells = 1:10. Similarly, lung-derived Gr1^−^ cells were incubated with lung-derived Gr1^+^ cells (control) or scaffold-derived Gr1^+^ cells (switched). Cells were co-cultured in vitro for two days at 37 °C. Then the frequencies of IFNγ^+^ and IL-17A^+^ CD4^+^ T cells and the frequencies of PRF1^+^GzmB^+^ CD8^+^ T cells were identified by flow cytometer.

### In vitro incubation of D-lung cells or their Gr1^−^ subpopulation with osteopontin and decorin proteins

Diseased lungs were retrieved from scaffold-free BALB/c mice bearing 4T1 tumor two weeks after orthotopic tumor inoculation and prepared to single-cell suspensions. Lung-derived Gr1^−^ cells were isolated by magnetic-activated cell sorting. Subsequently, unsorted lung cells or lung-derived Gr1^−^ cells were seeded into 24-well at a density of 2 × 10^6^ cell per mL per well without or with 50 ng/ml osteopontin and 50 ng/ml decorin. Cells were incubated for two days at 37 °C.

### Analysis of Kaplan–Meier curves of breast cancer patients

Kaplan–Meier curves were plotted by using an online survival analysis tool [http://kmplot.com/analysis/index.php?p=service]^60^. Auto select best cutoff was chosen to split patients to two cohorts in the analysis.

### Statistics and reproducibility

Data are presented as Mean ± Standard Error of the Mean (SEM). Animal studies were performed with at least two independent replicates of three to eight female BALB/c mice per group. Comparison between two groups was completed using Student’s two-tailed *t* test when not otherwise specified. Fisher’s exact test was used to analyze the metastasis *vs*. no metastasis categorical data in Fig. 3e. Bioluminescence data of lung metastases was not normally distributed, so the non-parametric Mann–Whitney test was used to assess *p*-values. *p*-values for the survival rate difference between scaffold-bearing mice and scaffold-free mice that received the surgery to remove the primary tumor at the same time were assessed from Mantel-Cox test. *p* values in the Kaplan–Meier curves were calculated with Mantel-Cox test. A *p* value of <0.05 was considered to indicate statical significance. No data were excluded from the analyses. Animals were allocated randomly.

### Reporting summary

Further information on research design is available in the Nature Portfolio Reporting Summary linked to this article.

### Supplementary information

Supplementary Information

Reporting Summary

### Source data

Source Data

## Data Availability

All data are available in the main text or the supplementary information. Source data, including data for Open array and Cytokine array, are provided with this paper. The single-cell RNA sequencing data generated in this study have been deposited in the ArrayExpress repositories under accession code E-MTAB-8503. The data for Kaplan–Meier curves presented in this study are available in [https://kmplot.com/analysis/]. The remaining data are available within the Article, Supplementary Information or Source Data file. Source data are provided with this paper.
