# Supplementary material for: A synthetic metastatic niche reveals antitumor neutrophils drive breast cancer metastatic dormancy in the lungs
Source: Nat Commun. 2023 Aug 8;14:4790. doi: 10.1038/s41467-023-40478-5 (PMC10409732; doi:10.1038/s41467-023-40478-5)
Supplement: Supplementary file 1 — Supplementary Information [file 41467_2023_40478_MOESM1_ESM.pdf]

Supplementary information for

A synthetic metastatic niche reveals antitumor neutrophils drive breast cancer metastatic dormancy in the lungs

Jing Wang[1, 2], Ramon Ocadiz-Ruiz[1], Matthew S. Hall[1], Grace G. Bushnell[1], Sophia M. Orbach[1], Joseph T. Decker[1, 3], Ravi M. Raghani[1], Yining Zhang[4], Aaron H. Morris[1, 5], Jacqueline S. Jeruss* [1, 6], Lonnie D. Shea* [1, 4, 6]

[1]Department of Biomedical Engineering, University of Michigan, Ann Arbor, MI, USA.

[2]Chemical and Biological Engineering Department, Iowa State University, Ames, IA, USA.

[3]Department of Cariology, Restorative Sciences, and Endodontics, University of Michigan School of Dentistry, Ann Arbor, MI, USA.

[4]Department of Chemical Engineering, University of Michigan, Ann Arbor, MI, USA.

[5]Rogel Cancer Center, University of Michigan, Ann Arbor, MI, USA.

[6]Department of Surgery, University of Michigan, Ann Arbor, MI, USA.

[*]Corresponding authors. Email: ldshea@umich.edu, jjeruss@umich.edu.

This pdf file includes Figures S1-S14 and Tables S1-S4.

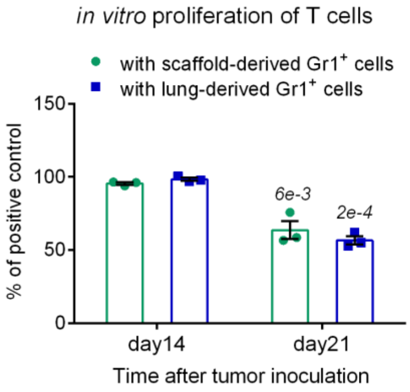

Fig. S1. Influences of Gr1+ cells derived from diseased scaffolds and lungs of BALB/c mice bearing 4T1 tumor at day 14 or day 21 after tumor inoculation on suppressing *in vitro* proliferation of naïve T cells. Data are shown as Mean ± SEM and *p* values are from student's two-tailed *t* test (day 21 *vs*. day 14). n = 3.

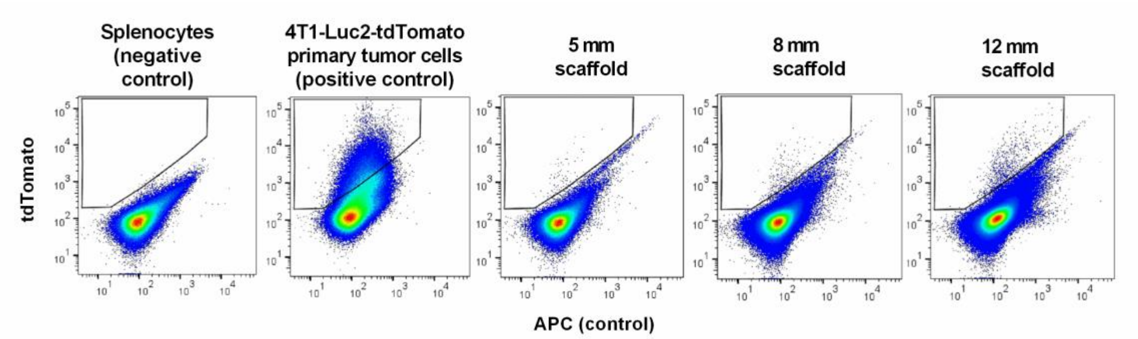

Fig. S2. Representative density plots for tdTomato+ 4T1 cancer cells migrating from the primary tumor to scaffold implants with different sizes in BALB/c mice bearing 4T1-Luc2-tdTomato tumor. Tissues were retrieved from mice two weeks after orthotopic inoculation of tumor cells, prepared to single cell suspensions, and then measured by flow cytometer. Splenocytes and primary tumor cells were negative and positive controls, respectively.

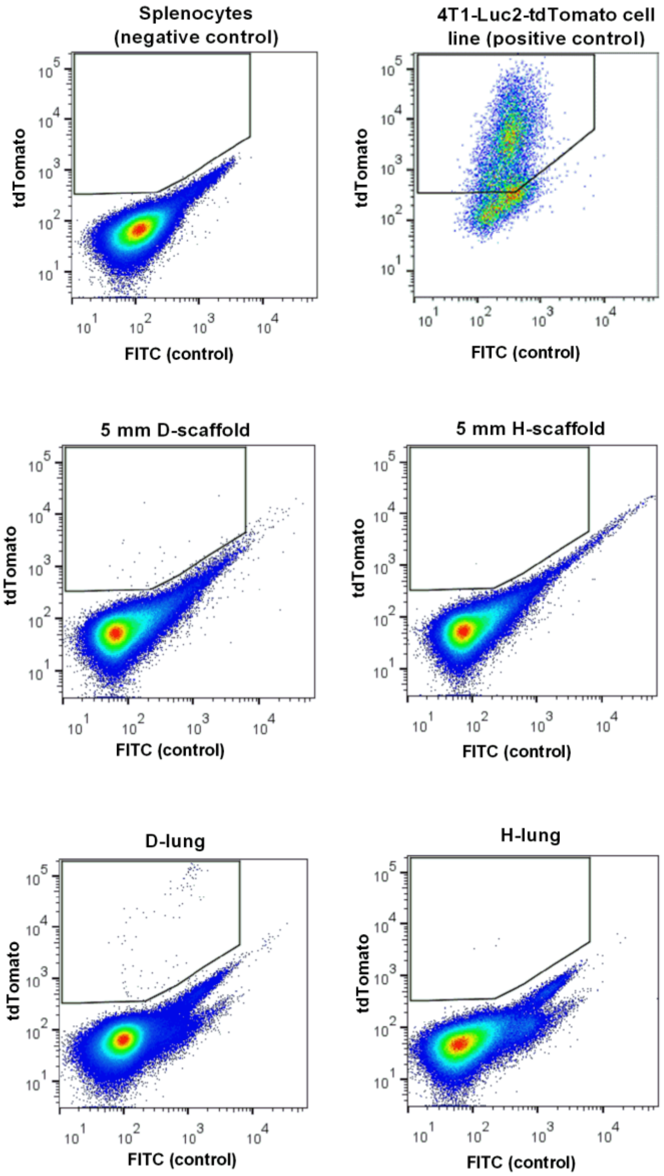

Fig. S3. Representative density plots for tdTomato$^+$ 4T1 cancer cells migrating to scaffold implants and lungs in tumor-free healthy BALB/c mice or diseased BALB/c mice bearing non-fluorescent 4T1 tumor. 4T1-Luc2-tdTomato cells were administrated through intracardiac injection.

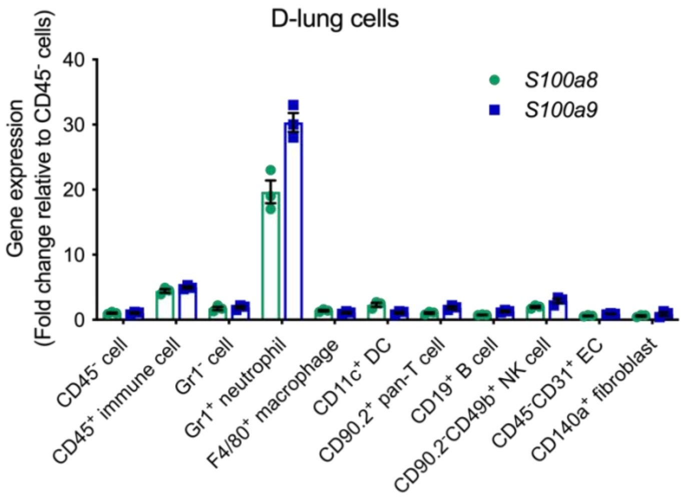

Figure S4. Expression of *S100a8* and *S100a9* genes in cell subsets of diseased lungs derived from 4T1-bearing mice. Cells were isolated by magnetic-activated cell sorting and the gene expression was analyzed by qRT-PCR. EC: endothelial cell. Data are shown as Mean ± SEM. n = 3.

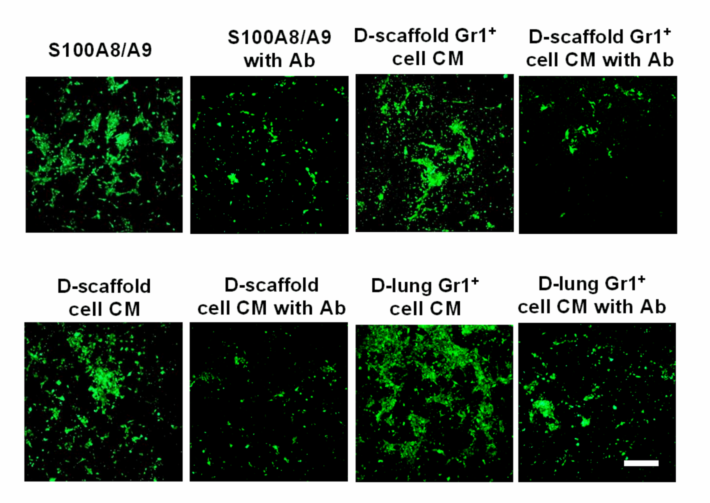

Fig. S5. Images of 4T1 cells transmigrating towards the S100A8/A9 recombinant proteins or conditioned media (CM) without or with supplementing anti-S100A8/A9 antibodies (Ab). Scale bar = 150 µm.

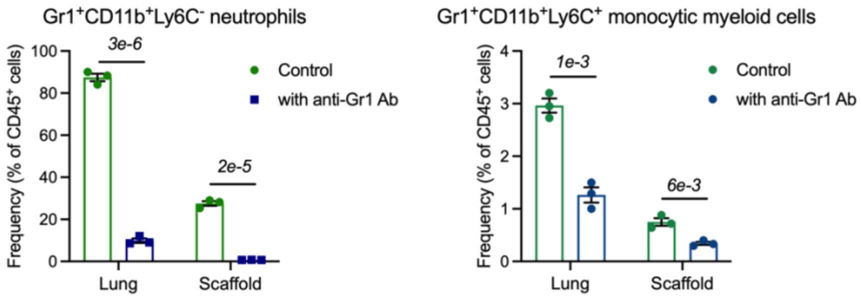

Fig. S6. Frequencies of Gr1$^+$CD11b$^+$Ly6G$^+$Ly6C$^-$ granulocytic (neutrophils) and Gr1$^+$CD11b$^+$Ly6G$^-$Ly6C$^+$ monocytic myeloid cells in the diseased lungs and scaffolds of 4T1-bearing BALB/c mice after mice were administrated with anti-Gr1 antibodies (Ab). Data are shown as Mean ± SEM and p values are from student's two-tailed t test. n =3.

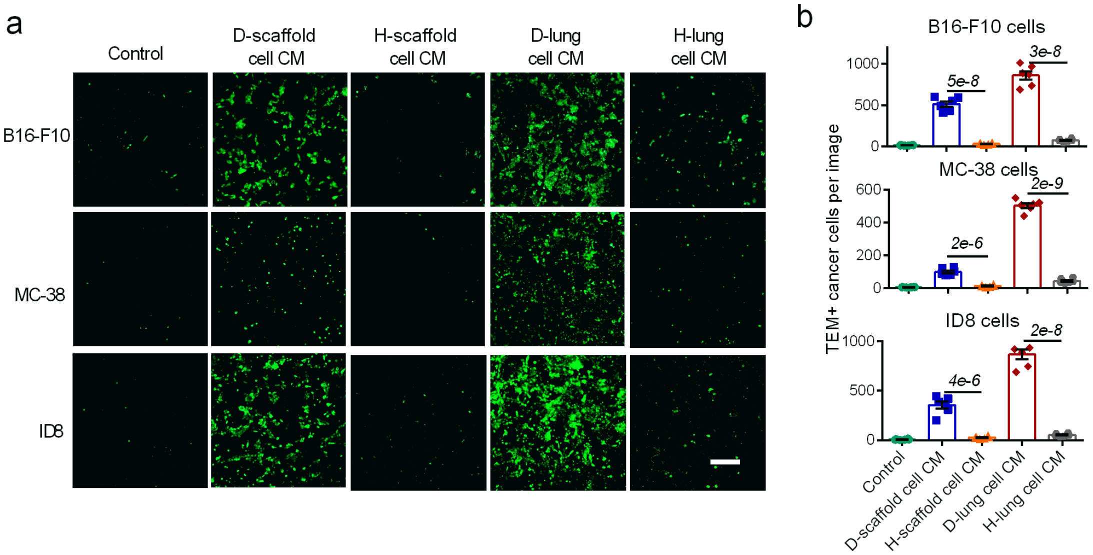

Fig. S7. *In vitro* extravasation of B16F10 murine melanoma cells, MC38 murine colon cancer cells and ID8 murine ovarian cancer cells to media conditioned by tissues derived from tumor-free or 4T1-bearing BALB/c mice. H: healthy; D: diseased. (**a**) Images of TEM$^+$ cancer cells, and (**b**) the quantification. Scale bar = 150 µm. CM: conditioned media; TEM: trans-endothelial migration. Data are shown as Mean ± SEM and p values are from student's two-tailed t test. n = 6 for (b).

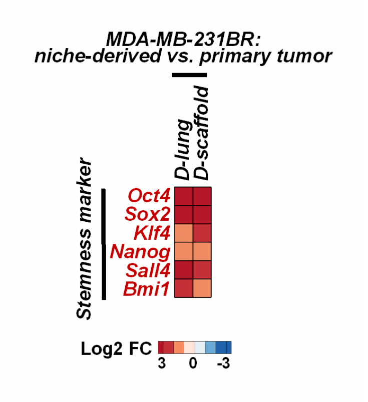

Fig. S8. MDA-MB-231BR (231BR) human breast cancer cells isolated from diseased lungs or scaffolds of NSG mice bearing 231BR tumor overexpressed stemness-associated markers compared to parental cells derived from the primary tumor.

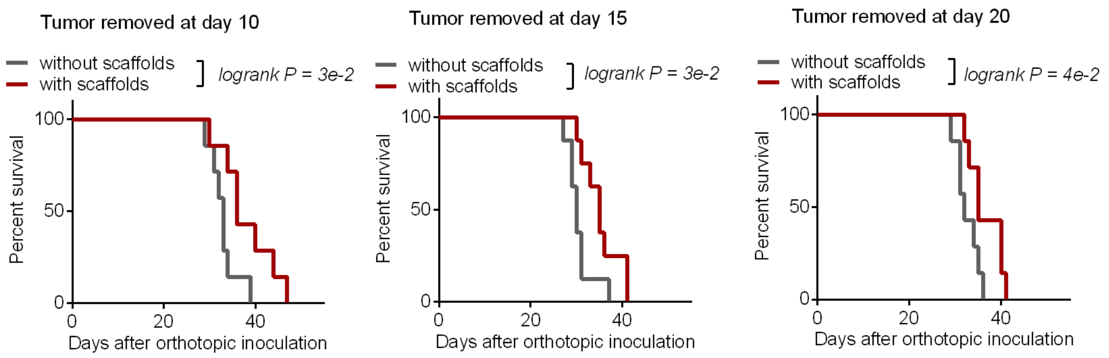

Fig. S9. Survival rates of scaffold-free and scaffold-bearing BALB/c mice after 4T1 primary tumors were resected at day 10, day 15, or day 20 after orthotopic tumor inoculation. *p* values are from Mantel-Cox test. n = 7 for groups receiving tumor resection surgery at day 10 or day 20 after tumor inoculation. n = 8 for groups receiving tumor resection surgery at day 15 after tumor inoculation.

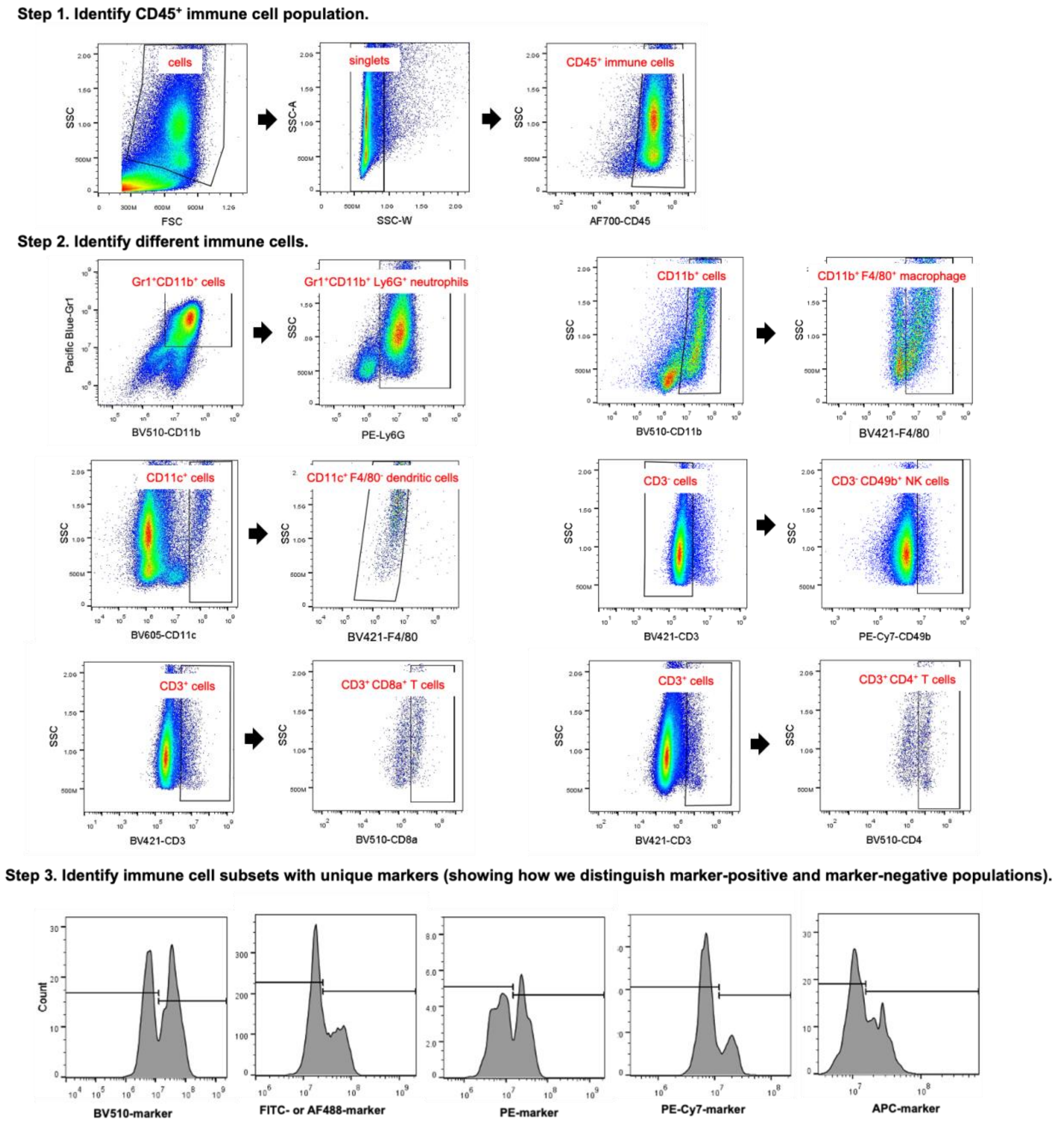

Fig. S10. Flow cytometric gating strategies to identify different immune subsets. There were three steps to identify the cell subsets shown in Fig. 4a. First (step 1), after identifying cell population with FSC and SSC, we used CD45 antibodies to distinguish $CD45^+$ immune cells from non-immune cells. Second (step 2), we employed surface markers to distinguish different immune cell populations among the $CD45^+$ population, including neutrophils, macrophages, dendritic cells, NK cells, $CD8^+$ T cells, and $CD4^+$ T cells. Last (step 3), for each immune cell population, we further employed surface or intracellular markers to distinguish cell subsets with different phenotypes. These markers were attached with fluorophores, including BV510, FITC/AF488, PE, PE-Cy7, or APC. We prepared unstained and single stained samples as controls to distinguish marker-positive and marker-negative populations in every flow cytometric

experiment. For example, to identify the frequency of N1 neutrophils, after gating cell population according to FSC and SSC, we first (step 1) gated $CD45^+$ immune cells with AF700-CD45 antibodies, and then (step 2) among the immune cells, we gated $Gr1^+CD11b^+Ly6G^+$ neutrophils with Pacific Blue-Gr1, BV510-CD11b, and PE-Ly6G antibodies, and last (step 3) among the neutrophils, we gated $NOS2^+TNF\alpha^+$ N1 neutrophils with PE-Cy7-NOS2 and FITC-TNFα antibodies. Details are in the Method section.

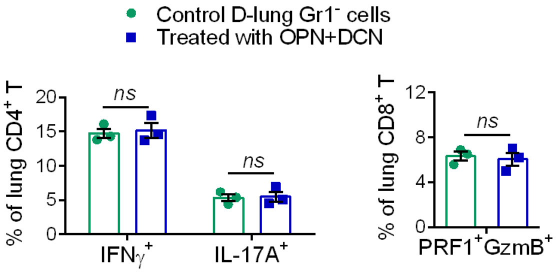

Fig. S11. Flow cytometric analysis of phenotypic changes in $Gr1^-$ D-lung cells after incubating with recombinant osteopontin (OPN) and decorin (DCN) proteins *in vitro*. Diseased lungs were derived from BALB/c mice bearing 4T1 tumor. Data are shown as Mean ± SEM and *p* values are from student's two-tailed *t* test. n = 3.

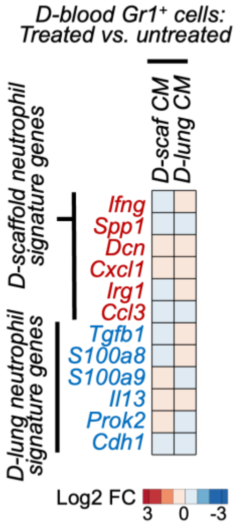

Fig. S12. The polarization phenotype of D-blood-derived Gr1$^+$ cells was not changed after they were incubated with media conditioned by diseased scaffold or lungs *in vitro*. Diseased blood, scaffolds, and lungs were derived from BALB/c mice bearing 4T1 tumor. CM: conditioned media.

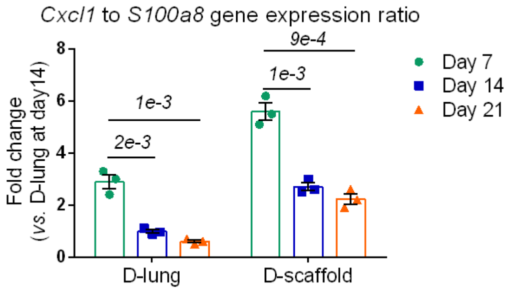

Fig. S13. The *Cxcl1*-to-*S100a8* gene expression ratio in diseased lungs and scaffolds of 4T1-bearing BALB/c mice as a function of time after tumor inoculation. Data are shown as Mean ± SEM and p values are from student's two-tailed t test. n = 3.

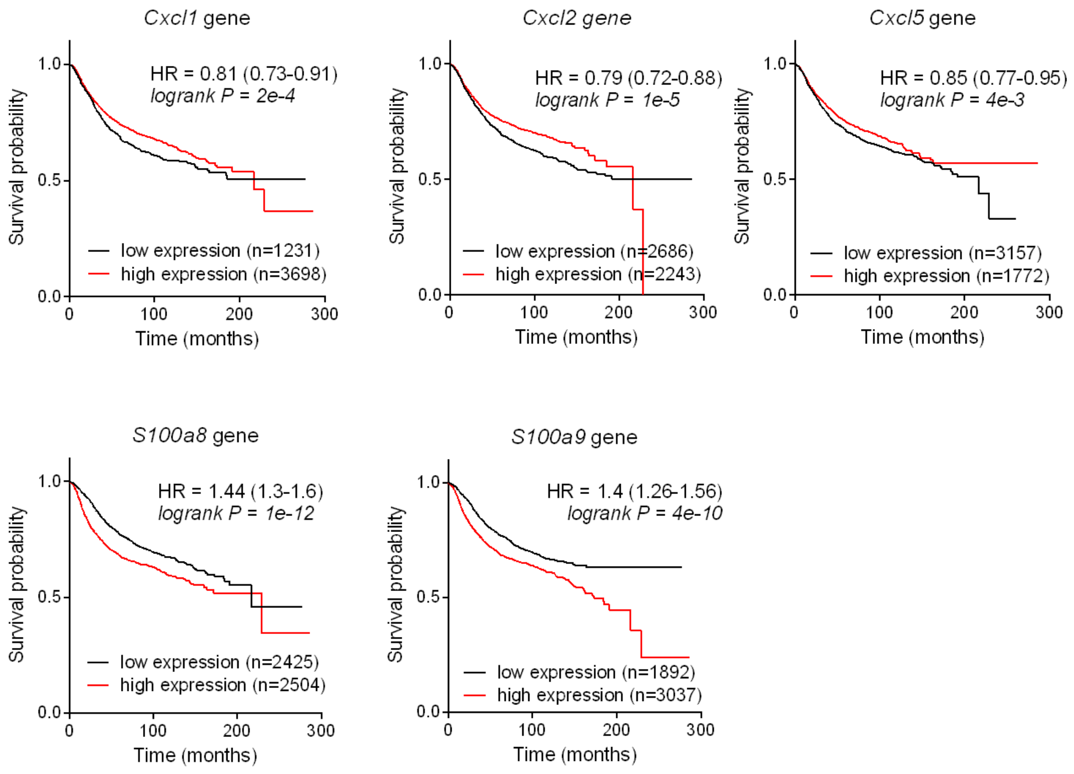

Fig. S14. Kaplan-Meier curves displaying the survival probability of breast cancer patients with low (black) or high (red) expression of genes for N1 chemoattractants (*Cxcl1*, *Cxcl2*, *Cxcl5*) or genes for N2 chemoattractants (*S100a8*, *S100a9*). n = number of patients with available clinical data. HR: hazard ratio. *p* values are from Mantel-Cox test.

Table S1. Microbeads or kits used in magnetic-activated cell sorting to isolate different cell populations.

**Cell types and markers**	**Kits**
CD45^{+} leukocytes and CD45^{-} cells	CD45 microbeads, mouse
Gr1^{+} myeloid cells and Gr1^{-} cells	myeloid-derived suppressor cell isolation kit, mouse
Ly6G^{+} neutrophils	myeloid-derived suppressor cell isolation kit, mouse
Gr1^{-}Ly6C^{+} monocytes	myeloid-derived suppressor cell isolation kit, mouse
F4/80^{+} macrophages	F4/80 microbeads ultrapure, mouse
CD11c^{+} dendritic cells	CD11c microbeads ultrapure, mouse
CD90.2^{+} pan-T cells	CD90.2 microbeads, mouse
CD19^{+} B cells	CD19 microbeads, mouse
CD90.2^{-}CD49b^{+} NK cells	CD90.2 microbeads and CD49b microbeads, mouse
CD45^{-}CD31^{+} endothelial cells	CD45 microbeads and CD31 microbeads, mouse
CD140a^{+} fibroblasts	CD140a (PDGFRα) microbead kit, mouse

Table S2. Antibodies used to analyze the frequencies of different immune cells in flow cytometry.

Panel #	BV421 405/420	BV510 405/520	BV605 405/610	FITC 488/520	PE 488/576	PE-Cy7 566/778	APC 652/657	AF700 702/723
1	PB-Gr1	BV510-CD11b		FITC-Ly6C	PE-Ly6G	PE-Cy7-F4/80	APC-CD11c	AF700-CD45
2	PB-CD19	BV510-CD4		FITC-CD8a		PE-Cy7-CD49b	APC-CD3	AF700-CD45

PB: Pacific Blue. BV: Brilliant Violet. AF: Alexa Fluor. Panel #1 distinguished Gr1^{+}CD11b^{+} myeloid cells, neutrophils, monocytes, macrophages and DCs, and panel #2 distinguished B cells, CD4^{+} T cells, CD8^{+} T cells, and NK cells.

Table S3. Antibodies used to analyze the phenotypes of different immune cells in flow cytometry.

Panel #	BV421 405/420	BV510 405/520	BV605 405/610	FITC 488/520	PE 488/576	PE-Cy7 566/778	APC 652/657	AF700 702/723
1	PB-Gr1	BV510-CD11b	BV605-IFNγ	FITC-TNFα	PE-Ly6G	PE-Cy7-NOS2		AF700-CD45
2	PB-Gr1	BV510-CD11b		AF488-CD206	PE-Ly6G		APC-IL10	AF700-CD45
3	PB-F4/80	BV510-CD11b	BV605-IFNγ	FITC-MHCII		PE-Cy7-NOS2		AF700-CD45
4	PB-F4/80	BV510-CD11b		AF488-CD206		PE-Cy7-Arg1		AF700-CD45
5	PB-F4/80		BV605-CD11c	FITC-MHCII			APC-CD86	AF700-CD45
6	PB-F4/80	BV510-CD11b	BV605-CD11c	FITC-CD103			APC-ESAM	AF700-CD45
7	BV421-CD3	BV510-CD4			PE-IFNγ	PE-Cy7-IL-17A	APC-TCRγ/δ	AF700-CD45
8	BV421-CD3	BV510-CD11b		AF488-CD27		PE-Cy7-CD49b		AF700-CD45
9	BV421-CD3	BV510-CD8a		FITC-TNFα	PE-IFNγ	PE-Cy7-CD49b		AF700-CD45
10	BV421-CD3	BV510-CD8a			PE-TRAIL	PE-Cy7-CD49b	APC-Fas	AF700-CD45
11	BV421-CD3	BV510-CD8a		FITC-GzmB	PE-PRF1	PE-Cy7-CD49b		AF700-CD45

Panels #1 and #2 analyzed N1 and N2 phenotypes of neutrophils, respectively. Panels #3 and #4 analyzed M1 and M2 phenotypes of macrophages, respectively. Panel #5 analyzed activation markers on DCs while panel #6 analyzed the cDC1 and cDC2 subsets of DCs. Panel #7 analyzed T cell subsets that overproduced IFNγ or IL-17A. Panel #8 analyzed maturation markers on NK cells. Panels #9, #10 and #11 analyzed the cytotoxicity of effector cells ($CD8^+$ T cells and NK cells) in terms of the expression of IFNγ, TNFα, Fas, TRAIL, perforin (PRF1), and granzymeB (GzmB).

Table S4. Antibodies used to analyze the frequency of CXCL1^{+} or S100A8^{+} cells in flow cytometry.

Panel #	BV421 405/420	BV510 405/520	BV605 405/610	FITC 488/520	PE 488/576	PE-Cy7 566/778	AF647 652/657	AF700 702/723
1	PB-Gr1	BV510-CD11b		AF488-S100A8	PE-Ly6G		AF647-CXCL1	AF700-CD45
