# Supplementary material for: A synthetic metastatic niche reveals antitumor neutrophils drive breast cancer metastatic dormancy in the lungs
Source: Nat Commun. 2023 Aug 8;14:4790. doi: 10.1038/s41467-023-40478-5 (PMC10409732; doi:10.1038/s41467-023-40478-5)
Supplement: Supplementary file 2 — Reporting Summary [file 41467_2023_40478_MOESM2_ESM.pdf]

nature portfolio

Corresponding author(s): Lonnie Shea, Jacqueline Jeruss

Last updated by author(s): 2023-7-16

Reporting Summary

Nature Portfolio wishes to improve the reproducibility of the work that we publish. This form provides structure for consistency and transparency in reporting. For further information on Nature Portfolio policies, see our Editorial Policies and the Editorial Policy Checklist.

Statistics

For all statistical analyses, confirm that the following items are present in the figure legend, table legend, main text, or Methods section.

n/a	Confirmed	
☐	☒	The exact sample size (n) for each experimental group/condition, given as a discrete number and unit of measurement
☐	☒	A statement on whether measurements were taken from distinct samples or whether the same sample was measured repeatedly
☐	☒	The statistical test(s) used AND whether they are one- or two-sided<br>*Only common tests should be described solely by name; describe more complex techniques in the Methods section.*
☐	☒	A description of all covariates tested
☐	☒	A description of any assumptions or corrections, such as tests of normality and adjustment for multiple comparisons
☐	☒	A full description of the statistical parameters including central tendency (e.g. means) or other basic estimates (e.g. regression coefficient) AND variation (e.g. standard deviation) or associated estimates of uncertainty (e.g. confidence intervals)
☐	☒	For null hypothesis testing, the test statistic (e.g. F, t, r) with confidence intervals, effect sizes, degrees of freedom and P value noted<br>*Give P values as exact values whenever suitable.*
☒	☐	For Bayesian analysis, information on the choice of priors and Markov chain Monte Carlo settings
☒	☐	For hierarchical and complex designs, identification of the appropriate level for tests and full reporting of outcomes
☒	☐	Estimates of effect sizes (e.g. Cohen's d, Pearson's r), indicating how they were calculated

Our web collection on statistics for biologists contains articles on many of the points above.

Software and code

Policy information about availability of computer code

Data collection	Flow Cytometry data was collected from Bio-Rad ZE5 cell analyzer with Everest software. Cytokine Array data was collected from Bio-Rad ChemiDoc with image lab touch software. Single-cell RNA sequencing data was collected were from the Illumina HiSeq 4000 sequencer.
Data analysis	Flow Cytometry Data was analyzed by FlowJo_V10. Cytokine Array Data was analyzed by Image Lab 6.1. Single-cell RNA-sequencing data was aligned to the mm10 mouse genome and cell identities, phenotypes, and differential gene expression were determined using the Seurat pipeline (version 3) in R. All the data were plotted by Prism 6 or Origin 2022.

For manuscripts utilizing custom algorithms or software that are central to the research but not yet described in published literature, software must be made available to editors and reviewers. We strongly encourage code deposition in a community repository (e.g. GitHub). See the Nature Portfolio guidelines for submitting code & software for further information.

Data

Policy information about availability of data

All manuscripts must include a data availability statement. This statement should provide the following information, where applicable:

- Accession codes, unique identifiers, or web links for publicly available datasets
- A description of any restrictions on data availability
- For clinical datasets or third party data, please ensure that the statement adheres to our policy

All data generated or analysed during this study are included in this published article (and its supplementary information files), and they are are available from the corresponding author on reasonable request.

Human research participants

Policy information about studies involving human research participants and Sex and Gender in Research.

Reporting on sex and gender	not applicable
Population characteristics	not applicable
Recruitment	not applicable
Ethics oversight	not applicable

Note that full information on the approval of the study protocol must also be provided in the manuscript.

Field-specific reporting

Please select the one below that is the best fit for your research. If you are not sure, read the appropriate sections before making your selection.

[x] Life sciences [ ] Behavioural & social sciences [ ] Ecological, evolutionary & environmental sciences

For a reference copy of the document with all sections, see nature.com/documents/nr-reporting-summary-flat.pdf

Life sciences study design

All studies must disclose on these points even when the disclosure is negative.

Sample size	Groups of 3-5 mice were used for studying the distribution of tumor cells in the scaffolds. Groups of 8 mice were used for the survival study. No statistical analysis was performed to predetermine sample size but these are standard numbers for the field. No sample size calculations were performed. Sample size was determined to be adequate based on magnitude and consistency of measurable differences between groups.
Data exclusions	No data was excluded.
Replication	Flow cytometry, TRACER, trans-endothelial migration, ELISA, and PCR experiments were repeated at least three times. Samples from 3-5 mice were pooled and then analyzed by single-cell RNA sequencing or Cytokine Array. Two replicates were prepared for OpenArray data. All attempts at replication were successful.
Randomization	BALB/c or C57 mice were purchased from The Jackson laboratory and divided in groups of same age and sex randomly.
Blinding	Mice were homogenous in sex and age prior to grouping. Blinding is not relevant in this study. Animal samples were analysed using objective, standardized assays that include appropriate controls. There is no subjective assessment of the animals.

Reporting for specific materials, systems and methods

We require information from authors about some types of materials, experimental systems and methods used in many studies. Here, indicate whether each material, system or method listed is relevant to your study. If you are not sure if a list item applies to your research, read the appropriate section before selecting a response.

Materials & experimental systems

n/a	Involved in the study
	☒ Antibodies
	☒ Eukaryotic cell lines
☒	Palaeontology and archaeology
	☒ Animals and other organisms
☒	Clinical data
☒	Dual use research of concern

Methods

n/a	Involved in the study
☒	ChIP-seq
	☒ Flow cytometry
☒	MRI-based neuroimaging

Antibodies

Antibodies used	Alexa Fluor 488-labeled anti-mouse S100A8 antibody (clone 63N13G5, #27067) and Alexa Fluor 647-labeled anti-mouse CXCL1 monoclonal antibody (clone 1174A, #IC4532R) were from Novus Biologicals. PE-Cyanine7 iNOS monoclonal antibody (CXNFT, #25-5920-80) and PE-Cyanine7 Arginase 1 monoclonal antibody (#25-3697-80) were from Thermo Fisher Scientific, eBioscience. All the other antibodies used in flow cytometry were from Biolegend, including Pacific Blue™ anti-mouse Ly-6G/Ly-6C (Gr-1) antibody (RB6-8C5, #108429), Pacific Blue™ anti-mouse CD19 antibody (6D5, #115526), Brilliant Violet 421™ anti-mouse F4/80 antibody (BM8, #123131), Brilliant Violet 421™ anti-mouse CD3 antibody (17A2, #100227), Brilliant Violet 510™ anti-mouse/human CD11b antibody (M1/70, #101245), Brilliant Violet 510™ anti-mouse CD4 antibody (GK1.5, #100449), Brilliant Violet 510™ anti-mouse CD8a antibody (53-6.7, #100751), Brilliant Violet 605™ anti-mouse CD11c antibody (N418, #117333), Brilliant Violet 605™ anti-mouse IFN-γ antibody (XMG1.2, #505839), FITC anti-mouse Ly-6C antibody (HK1.4, #128005), FITC anti-mouse CD8a antibody (53-6.7, #100705), Alexa Fluor® 488 anti-mouse CD206 (MMR) antibody (C068C2, #141709), FITC anti-mouse I-A/I-E (MHC class II) antibody (M5/114.15.2, #107605), FITC anti-mouse CD103 antibody (2E7, #121419), Alexa Fluor® 488 anti-mouse/rat/human CD27 antibody (LG.3A10, #124221), FITC anti-human/mouse Granzyme B antibody (GB11, #515403), FITC anti-mouse CD4 antibody (H129.19, #130308), PE anti-mouse Ly-6G antibody (1A8, #127607), PE anti-mouse IFN-γ antibody (XMG1.2, #505807), PE anti-mouse CD253 (TRAIL) antibody (N2B2, #109305), PE anti-mouse Perforin antibody (S16009A, #154305), PE anti-mouse CD25 antibody (3C7, #101903), PE/Cyanine7 anti-mouse F4/80 Recombinant antibody (QA17A29, #157307), PE/Cyanine7 anti-mouse CD49b antibody (HMα2, #103517), PE/Cyanine7 anti-mouse IL-17A antibody (TC11-18H10.1, #506921), APC anti-mouse CD11c antibody (N418, #117309), APC anti-mouse CD3 antibody (17A2, #100235), APC anti-mouse CD95 (Fas) antibody (SA367H8, #152603), APC anti-mouse CD86 antibody (GL-1, #105011), APC anti-mouse IL-10 antibody (JES5-16E3, #505009), APC anti-mouse ESAM antibody (1G8/ESAM, #136207), APC anti-mouse TCR γ/δ antibody (GL3, #118115), and Alexa Fluor® 700 anti-mouse CD45 antibody (30-F11, #103127). Anti-CDKN2A/p16INK4a antibody was from AbCam (EPR20418, #ab211542). Antibodies were diluted following manufacturer's instructions.
Validation	All the used antibodies are commercially available and their specificity (species), functionality, and suggested dilution can be found from the website and addressed in the above column.

Eukaryotic cell lines

Policy information about cell lines and Sex and Gender in Research

Cell line source(s)	4T1 (non-fluorescent) metastatic murine breast cancer cell line, MDA-MB-231 human breast cancer cell line, and HUVEC (human umbilical vein endothelial cell) were from ATCC. 4T1-Luc2-tdTomato cell line was from Perkin Elmer. 67NR and 4T07 non-metastatic murine breast cancer cell lines were from Karmanos Cancer Institute at Wayne State University. EO771-GFP murine breast cancer cell line was kindly provided by the lab of Gary and Kathryn Luker in the center for molecular imaging at the University of Michigan. B16F10 murine melanoma cell line, MC38 murine colon carcinoma cell line, and ID8 murine ovarian cancer cell line were kindly provided by the lab of Weiping Zou in the department of surgery at the University of Michigan.
Authentication	Cell lines were obtained from and authenticated by vendors or scientific collaborators. B16F10, MC38, and ID8 were originally from ATCC. 4T1-Luc2-tdTomato was originally from Caliper.
Mycoplasma contamination	The cell lines were not contaminated by mycoplasma as determined by using the Lonza Mycoplasma Detection Kit.
Commonly misidentified lines (See ICLAC register)	None

Animals and other research organisms

Policy information about studies involving animals; ARRIVE guidelines recommended for reporting animal research, and Sex and Gender in Research

Laboratory animals	Female BALB/c mice (6-7 weeks) from The Jackson laboratory were used for the study of 4T1, 67NR, and 4T07 breast cancers. Female C57 wild type and Ifng-/- and Il17a/Il17f-/- mice (6-7 weeks) from The Jackson laboratory were used for the study of EO771 breast cancer. Mice were housed in cages that were well ventilated, softly lit and subject to a light dark cycle. Mouse rooms and cages were kept at a temperature range of 20-24 C with humidity at 40-60%.
Wild animals	This study did not involve wild animals.
Reporting on sex	Female mice were used.

Field-collected samples	No field collected samples were used in this study.
Ethics oversight	All animal studies were performed in accordance with institutional guidelines and protocols (PRO00009715) approved by the University of Michigan Institutional Animal Care and Use Committee.

Note that full information on the approval of the study protocol must also be provided in the manuscript.

Flow Cytometry

Plots

Confirm that:

- [x] The axis labels state the marker and fluorochrome used (e.g. CD4-FITC).
- [x] The axis scales are clearly visible. Include numbers along axes only for bottom left plot of group (a 'group' is an analysis of identical markers).
- [x] All plots are contour plots with outliers or pseudocolor plots.
- [x] A numerical value for number of cells or percentage (with statistics) is provided.

Methodology

Sample preparation	Single cell suspensions were obtained from the lungs, scaffolds, tumor and spleens of mice, and cell subsets were isolated by magnetic-activated cell sorting (MACS) following manufacturer's instructions..
Instrument	Bio-Rad ZE5 cell analyzer
Software	Everest
Cell population abundance	>10,000 cells were collected and analyzed for immunostaining experiments and >100,000 cells were analyzed to identify fluorescent tumor cell abundance in different tissues.
Gating strategy	Negative controls (spleen cells) and positive controls (fluorescent primary tumor cells) were used to gate the fluorescent tumor cells in the lungs and scaffolds. Unstained cells and single-stained cells were used as negative and positive controls, respectively, in the analysis of the frequency and phenotype of different immune cells in the lungs and scaffolds.

- [x] Tick this box to confirm that a figure exemplifying the gating strategy is provided in the Supplementary Information.
